# Catalytic mechanism underlying the regiospecificity of coumarin-substrate transmembrane prenyltransferases in Apiaceae

**DOI:** 10.1093/pcp/pcae134

**Published:** 2024-11-22

**Authors:** Junwen Han, Ryosuke Munakata, Hironobu Takahashi, Takao Koeduka, Mayumi Kubota, Eiko Moriyoshi, Alain Hehn, Akifumi Sugiyama, Kazufumi Yazaki

**Affiliations:** Laboratory of Plant Gene Expression, Research Institute for Sustainable Humanosphere, Kyoto University, Gokasho, Uji, Kyoto 611-0011, Japan; Laboratory of Plant Gene Expression, Research Institute for Sustainable Humanosphere, Kyoto University, Gokasho, Uji, Kyoto 611-0011, Japan; Faculty of Pharmaceutical Sciences, Tokushima Bunri University, Nishihama, Yamashiro-cho, Tokushima 770-8514, Japan; Graduate School of Sciences and Technology for Innovation, Yamaguchi University, 1677-1, Yoshida, Yamaguchi City, Yamaguchi 753-8511, Japan; Graduate School of Sciences and Technology for Innovation, Yamaguchi University, 1677-1, Yoshida, Yamaguchi City, Yamaguchi 753-8511, Japan; Laboratory of Plant Gene Expression, Research Institute for Sustainable Humanosphere, Kyoto University, Gokasho, Uji, Kyoto 611-0011, Japan; Université de Lorraine, INRAE, LAE, Nancy F54000, France; Laboratory of Plant Gene Expression, Research Institute for Sustainable Humanosphere, Kyoto University, Gokasho, Uji, Kyoto 611-0011, Japan; Laboratory of Plant Gene Expression, Research Institute for Sustainable Humanosphere, Kyoto University, Gokasho, Uji, Kyoto 611-0011, Japan

**Keywords:** Apiaceae, coumarin, *Pastinaca sativa*, membrane-bound prenyltransferase, regiospecificity, UbiA superfamily

## Abstract

Plant membrane-bound prenyltransferases (PTs) catalyze the transfer of prenyl groups to acceptor substrates, phenols, using prenyl diphosphates as the donor substrate. The presence of prenyl residues in the reaction products, prenylated phenols, is key to the expression of a variety of physiological activities. Plant PTs generally exhibit high specificities for both substrate recognition and prenylation sites, while the molecular mechanism involved in these enzymatic properties is largely unknown. In this study, we performed a systematic biochemical analysis to elucidate the catalytic mechanism responsible for the reaction specificity of plant PTs. Using two representative PTs, PsPT1 and PsPT2, from parsnip (*Pastinaca sativa*, Apiaceae), which differ only in the regiospecificity of the prenylation site, we performed domain swapping and site-directed mutagenesis of these PTs, followed by detailed enzymatic analysis combined with 3D modeling. As a result, we discovered the domains that control prenylation site specificity and further defined key amino acid residues responsible for the catalytic mechanism. In addition, we showed that the control mechanism of prenylation specificity revealed here is also highly conserved among coumarin-substrate PTs. These data suggest that the regulatory domain revealed here is commonly involved in prenylation regiospecificity in Apiaceae PTs.

## Introduction

To survive in harsh environments, plants have acquired the ability to biosynthesize a large number of secondary metabolites with a wide variety of chemical structures. These secondary metabolites produced by plants have also been utilized in various aspects of human life (Sen and Samanta 2015). Among them, prenylated phenolics comprise a group of ∼4000 metabolites that perform important physiological functions in plants, such as chemical defense against pests and diseases (Munakata and Yazaki 2024). As many prenylated compounds exhibit diverse biological activities, they have also been identified as active principles of various medicinal plants (Yazaki et al. 2009). Typical examples are shikonin, which has antimicrobial and anti-inflammatory activities, produced in the family Boraginaceae (Yazaki 2017); artepillin C, which has anti-tumor and anti-obesity activities, found in Asteraceae plants (Munakata et al. 2019); macluraxanthone B and daunochromenic acid, which have anti-HIV activities from *Maclura* and *Rhododendron* species, respectively (Groweiss et al. 2000, Saeki et al. 2018); and tetrahydrocannabinol, which has psychoactive activities from Cannabaceae plants (Amin and Ali 2019).

**Figure 1.**
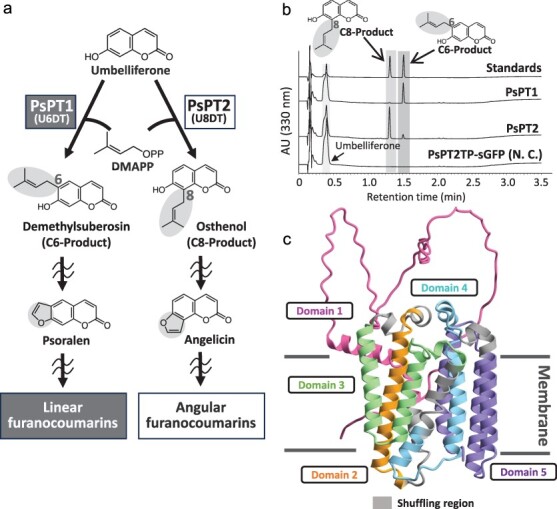
A pair of PTs from the apiaceous species parsnip. (a) Prenylation activities of PsPT1 and PsPT2. Umbelliferone and DMAPP are used as substrates. PsPT1 preferentially shows the U6DT activity to biosynthesize the C6-position product, demethylsuberosin. PsPT2 preferentially shows the U8DT activity to biosynthesize the C8-position product, osthenol. Demethylsuberosin and osthenol end up in different forms of furanocoumarins due to differences in prenylation sites. (b) UV chromatograms at 330 nm of UDT reaction mixtures of PsPT1 and PsPT2. The transit peptide (TP) region of PsPT2 fused to sGFP (PsPT2TP-sGFP) (Munakata et al. 2016) was used as a negative control. The UV chromatograms of PsPT1, PsPT2, and the negative control are shown at a comparable scale. (c) A 3D model diagram of PsPT1 built with AlphaFold2 (UniProt ID, AF-A0A0U1ZEE9-F1-model_v4) was visualized using CueMol2. The detailed information on domain configuration is given in Supplementary Fig. S1.

The presence of prenyl groups is critical for the exertion of these diverse functions, and prenyltransferases (PTs) are the biosynthetic enzymes responsible for transferring prenyl residues to the phenolic backbone (Munakata and Yazaki 2024). An important enzymatic feature of plant PTs is that they generally exhibit high specificities for both substrate recognition and prenylation sites (Yang et al. 2023, Ernst et al. 2024), which is unlikely for bacterial and fungal PTs, which are mostly soluble enzymes (Mori 2020). This specificity is the basis for plants to develop species-specific chemical defenses, such as the arms race relevant to furanocoumarins (Berenbaum and Feeny 1981). Furanocoumarins are divided into two groups, linear and angular types, and the former are biosynthesized as primary chemical defense compounds in some plant families such as Apiaceae and Rutaceae. The latter serves as a chemical barrier against specialist herbivores that can degrade linear furanocoumarins. The difference between linear and angular furanocoumarins is generated by the prenylation position of umbelliferone, a key intermediate of furanocoumarin biosynthesis, i.e. C6-prenylated and C8-prenylated umbelliferone are the direct precursors of linear and angular furanocoumarins, respectively (Munakata et al. 2016) (Fig. 1a). However, it is still unclear how plant PTs are able to catalyze such highly specific prenylation reactions. One of the reasons for this is that plant PTs belong to the UbiA superfamily, which has nine transmembrane alpha helices, making crystal structure analysis generally difficult.

In 2008, our laboratory isolated the first flavonoid-specific PT gene, *SfN8DT-1*, from the medicinal plant *Sophora flavescens* (Sasaki et al. 2008). This discovery was followed by the identification of an isoflavone-specific PT, SfG6DT, which shares 64% amino acid sequence identity with SfN8DT-1 (Sasaki et al. 2011). Focusing on the specific catalytic mechanisms of PTs, chimeric enzymes of PTs with different activities were generated and amino acid residues important for reaction specificity were analyzed. However, only a few chimeric PTs showed prenylation activity in the yeast expression system, and narrowing down the critical amino acid could not be achieved in the previous attempts (Sasaki et al. 2011). In Archaea, the first crystal structure of the phenolic substrate PT was reported, and two aspartate-rich motifs essential for prenylation activity were shown to trap prenyl diphosphate (Cheng and Li 2014, Huang et al. 2014), while these results could not elucidate the enzymatic specificities. A systematic attempt using our 28-plant PT collection to obtain high protein expression of PT in some yeast hosts was also unsuccessful. Thus, it remains to be elucidated how plant PTs acquire their substrate and product specificities, i.e. more knowledge is needed on the catalytic mechanism responsible for the high degree of reaction specificity of plant PTs.

The study of plant PTs has been intensively developed in recent years, and many genes and amino acid sequences of PTs have been reported. Taking advantage of this, we were able to select an optimal PT pair for the preparation of chimeric enzymes, which were functionally expressed in a plant host with high efficiency. To elucidate the catalytic mechanism responsible for the high reaction specificity of plant PTs, we undertook a multifaceted research combining the chimera enzyme studies, site-directed mutagenesis of PTs, and the latest 3D protein structure prediction techniques. We also investigated the conservation of the regulatory mechanism of enzyme function in coumarin-specific PTs in Apiaceae.

## Results

### Selection of PT pairs for chimeric enzymes

We tried to select the most suitable PT pairs for the preparation of chimeric enzymes to explain the catalyst specificity among the known plant PTs. We thoroughly searched for plant PTs in the published reports and collected a total of 60 plant PT sequences. After pairing each of these 60 PTs one-to-one, we obtained 1770 PT combinations. We arranged them in order of their amino acid sequence identity with each other and removed the pairs of PTs with the same reaction specificities or those without comparable biochemical data. This screening highlighted PsPT1 and PsPT2 from *Pastinaca sativa* in the Apiaceae (Munakata et al. 2016), which share the highest amino acid identity (71%) (Supplementary Table S1). These PTs commonly use dimethylallyl diphosphate (DMAPP) as a prenyl donor substrate and umbelliferone (7-hydroxycoumarin) as an acceptor substrate, whereas these PTs have different regiospecificities. PsPT1 introduces a prenyl residue at the C6 position of umbelliferone, resulting in the formation of demethylsuberosin. On the other hand, PsPT2 attaches a prenyl residue to the C8 position of umbelliferone leading to the synthesis of osthenol (Fig. 1a and b). Therefore, we selected PsPT1 and PsPT2 as the most suitable PT pairs to proceed with the domain-swapping experiment to analyze the regiospecificity of the PT reaction.

### Regiospecificity of the domain-swapped mutants

**Figure 2.**
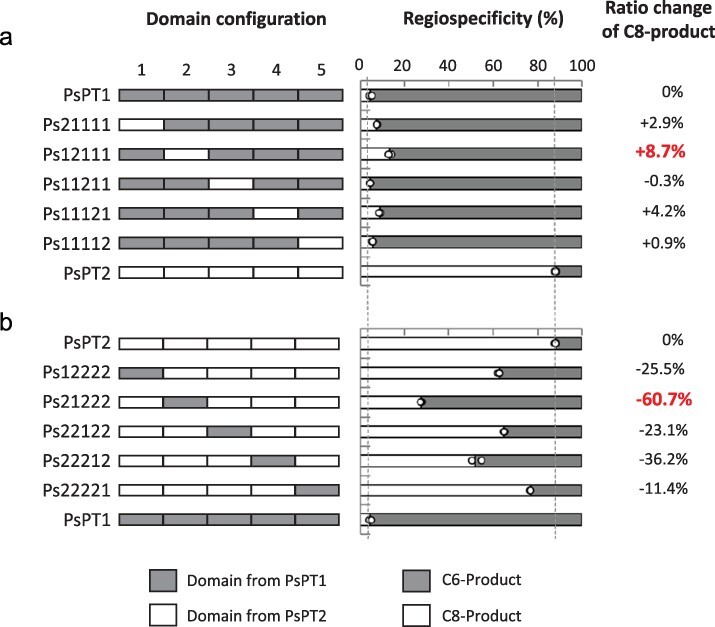
The regiospecificity of single domain-swapped chimeric enzymes of PsPT1 and PsPT2. (a) The regiospecificity of mutants generated by substitution of the PsPT1 sequence with a domain derived from PsPT2. (b) The regiospecificity of mutants generated by substitution of the PsPT2 sequence with a domain derived from PsPT1. The regiospecificity of each protein was assessed by independent triplicate reactions (*n* = 3). Domain configuration: gray bars, domains from PsPT1; white bars, domains from PsPT2. Regiospecificity: gray bars, prenylation product at the C6 position; white bar, prenylation product at the C8 position. Data of PsPT1 and PsPT2 are identical between (a) and (b). The regiospecificity of all domain-swapped mutants is shown in Supplementary Fig. S2.

For the domain-swapping experiments, four shuffling regions were selected based on the identical amino acid sequence segment of PsPT1 and PsPT2 to divide these two PTs into five domains (domains 1–5 from the N-terminus) (Fig. 1c, Supplementary Fig. S1). We then recombined each domain of PsPT2 with the corresponding domain of PsPT1 to generate serial domain-swapped PTs (Fig. 2a) and applied the same recombination in reverse (Fig. 2b). In this way, we generated a total of 30 different domain-swapped chimeric enzymes (Supplementary Fig. S2). For ease of description in this experiment, we use the numbers 1 and 2 to represent the domain from PsPT1 and the domain from PsPT2, respectively. For example, PsPT1 is referred to as Ps11111 in this article, and the same procedure was applied to other domain-swapped enzymes. For the expression of the recombinant enzymes, we chose *Nicotiana benthamiana* as the host organism because the success rate of functional expression is much higher than yeast for membrane-bound PTs of plant origin (Karamat et al. 2014). Due to the large number of chimeric enzymes to be analyzed, we have developed a simplified method for the preparation of crude enzymes containing membrane proteins to achieve a higher throughput than the original method of microsome preparation, using conventional centrifugation at 15 000 × *g* instead of ultracentrifugation to prepare the membrane protein fraction (see the Materials and Methods section). Only when the PT activity of a chimera enzyme was very low did we use the original ultracentrifuge preparation method. Using umbelliferone and DMAPP as substrates, we analyzed the PT activities of all chimera enzymes by HPLC and calculated the regiospecificity (%) (Fig. 2a, Supplementary Fig. S2), showing the chimera enzymes with a single domain substitution and arranged separately for comparison. The ratio change is expressed as the proportion of osthenol (C8-prenylated umbelliferone), which was calculated as follows: [% mutant C8-position product] − [% wt C8-position product], and the value of [% wt C8-position product] is 88.2% as shown in Fig. 2 and Supplementary Fig. S2. This value is used as a parameter to evaluate the prenylation site specificity. Among five domains, domain 2 gave the largest ratio change, i.e. domain 2 derived from PsPT1 shifted the ratio towards the C6-prenylated product (Ps21222, −60.7%), whereas that from PsPT2 shifted the ratio towards the C8-prenylated product (Ps12111, +8.7%). When PsPT1 was replaced by a single domain of PsPT2, we found that the single domain replacement of domain 2 caused the largest regiospecific changes (Fig. 2a). Conversely, when PsPT2 was replaced by a single domain of PsPT1, we observed more pronounced changes in the ratio change of the C8-prenylated product. In particular, a single substitution of domain 2 resulted in ∼60% regiospecific changes, which was significantly higher than the remaining four domains (Fig. 2b). The results of multiple domain substitutions are also shown in Supplementary Fig. S2. These data indicate that domains from PsPT2 have an obvious contribution to the formation of a higher proportion of osthenol in the chimera enzymes, while each domain has a different influence on regiospecificity. For example, only one domain of Ps12111, domain 2 from PsPT2, is present in the chimera enzyme, and its osthenol formation ratio is higher than that of Ps11222, which has three domains from PsPT2 (Fig. 2a, Supplementary Fig. S2). Therefore, we concluded that, irrespective of the sequence of PsPT1 or PsPT2, domain 2 is the one that has the greatest influence on the regiospecificity of prenylation.

### Regiospecificity in site-directed mutagenesis studies

**Figure 3.**
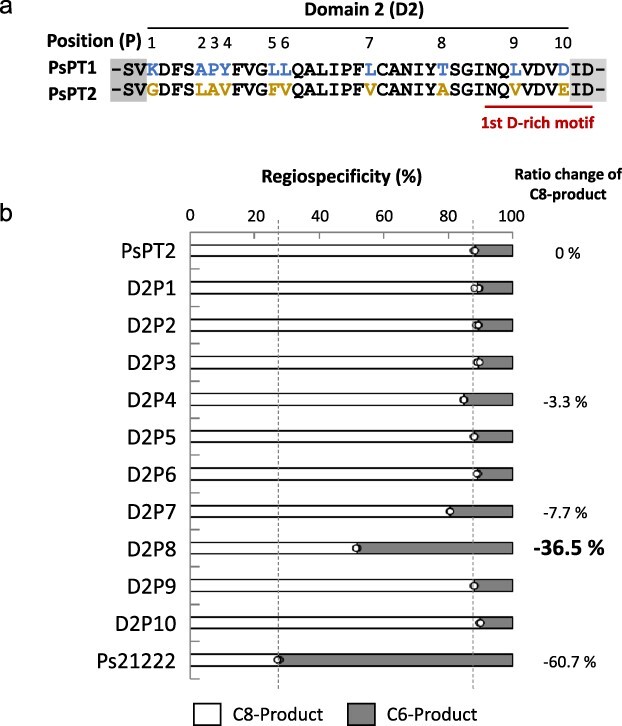
Introduction of point mutations of amino acids from PsPT1 into the sequence of domain 2 in PsPT2. (a) Ten mismatch sites between domain 2 of PsPT1 and PsPT2. (b) Point mutations were individually introduced into domain 2 of PsPT2 at 10-mismatch sites using the amino acids of PsPT1 to create 10 point mutants and their regiospecificities were confirmed by independent triplicate reactions (*n* = 3). White bar, prenylation product at the C8 position; gray bar, prenylation product at the C6 position. Data of PsPT2 and Ps21222 in Supplementary Fig. S2 are shown as controls.

We then tried to determine which amino acid in domain 2 is responsible for regulating the regiospecificity of these PTs. By aligning the domain 2 amino acid sequences of PsPT1 and PsPT2, we found 10 amino acid mismatches between them, referred to as domain 2 positions 1–10 (D2P1–D2P10) (Fig. 3a). We performed site-directed mutagenesis in PsPT2, introducing each amino acid found in PsPT1. By replacing the amino acids at these 10 different amino acid positions (D2P1–D2P10) of PsPT2 with the amino acids in PsPT1, 10 point mutant PTs were generated (PsPT2-D2P1 to PsPT2-D2P10) (Fig. 3b). The recombinant enzymes were prepared after transient expression in *N. benthamiana*, and the enzyme assay was performed in the same way as for the domain-substituted chimera enzymes mentioned earlier. As a result, we found that the mutation at the eighth amino acid position from the N-terminus of domain 2 [mutant enzyme PsPT2-D2P8(A/T)] gave a strongly altered regiospecificity. The ratio change of osthenol (C8-prenylated umbelliferone) was −36.5%. Comparing the influence of domain 2 shown in the domain-swapped enzyme Ps21222, this effect of the D2P8 mutant enzyme largely contributed to the regiospecificity of PT, i.e. almost 60% of the total effect of domain 2 is due to the D2P8 mutation (Fig. 3b). The importance of D2P8 is also supported by the threonine-to-alanine mutation at D2P8 of Ps21222, which showed a strong influence on regiospecificity (Supplementary Fig. S3). The effect on the regiospecificity was comparable between this point mutation and the domain 2 swapping for PsPT1 (Supplementary Fig. S4), although the change of the regiospecificity was smaller than for Ps21222.

We also introduced point mutations in domain 4, which has the second largest effect after domain 2. We aligned the domain 4 amino acid sequences of PsPT1 and PsPT2 and found that there are 17 amino acid differences between them in domain 4 (Supplementary Fig. S5). By replacing the amino acids at these 17 different amino acid positions of PsPT2 with the amino acids on PsPT1, we generated 17 point mutants (PsPT2-D4P1 to PsPT2-D4P17). While the reaction product was not detectable for PsPT2-D4P8(V/A), most of the remaining PT point mutants did not show clear changes in regiospecificity. The proportion of osthenol in the two PT point mutants, PsPT2-D4P10(I/F) and PsPT2-D4P12(A/G), appeared to be reduced by 9.4% and 5.5%, respectively (Supplementary Fig. S5). This suggests that some amino acids in domain 4 also influence the regiospecificity of PT, although their influence is much less than that of D2P8.

The contribution of the D2P8 position to the control of domain 2 regiospecificity was prominent, while this single amino acid substitution does not explain the entire influence of domain 2. Some other amino acid may contribute to the regulation of regiospecificity, while its single mutation does not give strong effects. We then picked up two mutations, one from domain 2 D2P4 and other from domain 4 D4P10, to make double mutants together with D2P8(A/T) for PsPT2. *In vitro* assays of the resulting double mutants showed that both D2P4 and D4P10 mutations were effective on the regiospecificity of PsPT2-D2P8(A/T) (Supplementary Fig. S6). Unfortunately, despite several attempts, we were unable to construct the double mutant for D2P7 and D2P8.

### Type of amino acid residues influencing regiospecificity

We next investigated how regiospecificity is affected by different amino acids at D2P8. In our mutagenesis between threonine and alanine at D2P8, the regiospecificity of Ps21222 was more variable than that of PsPT1 or PsPT2 (Fig. 3, Supplementary Figs S3 and S4), suggesting that the effect of different mutations can be accurately assessed using Ps21222 as a backbone. Eighteen amino acids other than the threonine of PsPT1 and the alanine of PsPT2 were introduced at the D2P8 position of Ps21222 to generate 18 point mutant enzymes (Supplementary Fig. S3). Among them, only the point mutations of the three amino acids cysteine, glycine, and valine gave detectable levels of reaction products, and no clear reaction products were detected in the remaining mutant enzymes. Among these three, the proportion of C8-position product reached ∼76.9% in the cysteine-introduced mutant, which was 41.9% higher than the original Ps21222 and 5.6% higher than that of alanine-introduced mutant PT (Supplementary. Fig. S3). We also introduced cysteine, glycine, or valine at position D2P8 of PsPT2. The results showed that the contribution of these residues to the regiospecificity of PsPT2 was similar to that of Ps21222. In particular, the cysteine mutation introduced at the D2P8 position of PsPT2 increased the amount of C8-position product by 5.5%, which was above the upper limit of the amount of product observed in natural PsPT2 (Supplementary Fig. S7). We tested the cysteine mutation at the D2P8 position of PsPT1, but its effect was much smaller than that caused by the introduction of alanine (Supplementary Fig. S4), suggesting that the cysteine at D2P8 changes the regiospecificity towards C8 prenylation with the help of different residues outside domain 2.

Using representative mutant enzymes, we performed detailed kinetic studies and calculated their apparent *K_m_* values (Table 1, Supplementary Fig. S8). PsPT1 shows *K_m_* values of 5 ± 1 μM and 2.7 ± 0.6 μM for DMAPP and umbelliferone, respectively, in the formation of the major C6-position product, and the *K_m_* values are almost similar for the minor C8-position product. However, the D2P8 mutation (threonine to alanine) increased the *K_m_* value for umbelliferone >10-fold for both products (C6-position product, 47 ± 7 µM; C8-position product, 35 ± 7 µM), whereas that for DMAPP was not strongly affected. PsPT2 formed the major C8-position product with *K_m_* values of 7.6 ± 0.8 μM and 10 ± 1 μM for DMAPP and umbelliferone, respectively, whereas the D2P8 mutation (alanine to threonine) caused a strong increase in the *K_m_* values for both substrates, i.e. 100 ± 20 μM (DMAPP) and 50 ± 4 μM (umbelliferone). In general, the regiospecificity of PT could involve the interactions of the enzyme protein with both prenyl donor and acceptor substrates. In our kinetic analysis, PsPT1 and PsPT2 commonly affected their apparent *K_m_* values for the prenyl acceptor, suggesting that the interaction with the acceptor substrate mainly regulates the regiospecificity of PsPTs. Interestingly, the D2P8 mutation from alanine to cysteine did not increase the *K_m_* values, showing that the regiospecificity is enhanced while maintaining affinity for both substrates.

**Table 1.** Apparent *K_m_* values of PsPT1/2 and their mutants

	Demethylsuberosin		Osthenol	
Product	(C6-position product)		(C8-position product)	
Substrate	DMAPP	Umbelliferone	DMAPP	Umbelliferone
PsPT1	5 ± 1	2.7 ± 0.6	5 ± 1	3 ± 2
PsPT1-D2P8 (T/A)	4.4 ± 0.8	47 ± 7	4.0 ± 0.8	35 ± 7
PsPT2	7.2 ± 0.8	10.5 ± 0.9	7.6 ± 0.8	10 ± 1
PsPT2-D2P8 (A/T)	90 ± 20	48 ± 3	100 ± 20	50 ± 4
PsPT2-D2P8 (A/C)	4.2 ± 0.5	8 ± 2	2.4 ± 0.2	4.7 ± 0.6

Data are presented as mean ± standard error (*n* = 3).

### 3D modeling analysis

**Figure 4.**
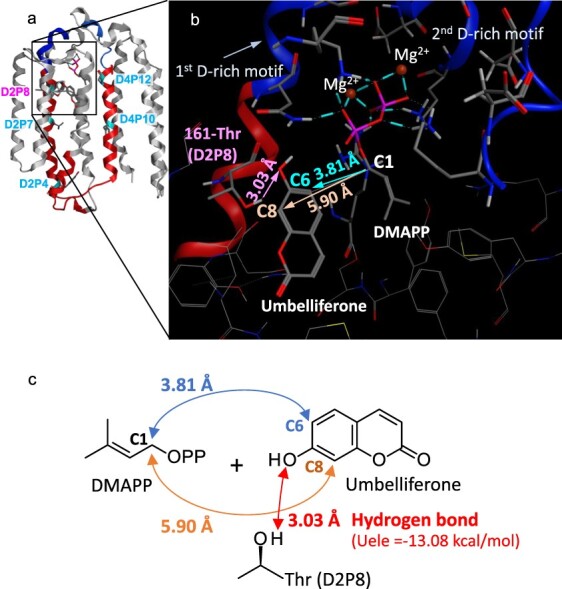
A 3D model of substrate-bound PsPT1. The overall structure (a) and the catalytic pocket (b) of the most appropriate ligand configuration obtained through docking simulation analysis (Supplementary Table S2). The distance between C1 of DMAPP and C6 of umbelliferone is related to the U6DT activity, and the distance between C1 of DMAPP and C8 of umbelliferone is related to the U8DT activity. (c) Model diagram of the interaction between the D2P8 position (161-Thr) of PsPT1 and umbelliferone. A hydrogen bond (Uele = −13.08 kcal/mol) between umbelliferone and 161-Thr is proposed.

Since crystallography is not feasible for the plant PTs, we used AlphaFold2 to construct 3D models of PsPT1 and PsPT2 (Jumper et al. 2021), and AseDock from Molecular Operating Environment (MOE) to perform simulation analysis of the two ligands (umbelliferone and DMAPP) of PsPT1 and PsPT2. We then found that the ligand configuration was consistent with the umbelliferone 6-dimethylallyltransferase (U6DT) activity of PsPT1 and the umbelliferone 8-dimethylallyltransferase (U8DT) activity of PsPT2 (Supplementary Table S2). In the 3D model of PsPT1, the distance between the C1 position of DMAPP and the C6 position of umbelliferone is 3.81 Å, which is shorter than the distance of 5.90 Å between the C1 position of DMAPP and the C8 position of umbelliferone, and this model is therefore considered to be a reasonable model for U6DT (Fig. 4a, b). The distance between the hydroxyl oxygen atom of umbelliferone and the hydroxyl hydrogen atom of threonine at position D2P8 is 3.03 Å. In addition, the Coulombic energy (Uele) between these two atoms is about −13.08 kcal/mol, which is thought to be sufficient for hydrogen bonding, so threonine at position D2P8 is adjacent to umbelliferone and is likely to be able to interact with it (Fig. 4c). We then predicted amino acid positions that could interact with DMAPP and umbelliferone in the model of PsPT1 by MOE. Among the 17 predicted amino acid positions, it was found that the only position where the amino acid residue differs between PsPT1 and PsPT2 is the D2P8 position (Supplementary Figs S1 and S9A).

On the other hand, in the 3D model of PsPT2, the distance between the C1 position of DMAPP and the C8 position of umbelliferone is 3.63 Å, which is shorter than the distance of 5.66 Å between the C1 position of DMAPP and the C6 position of umbelliferone; therefore, this model is considered to be a model of U8DT (Supplementary Fig. S10). The distance between the hydroxyl oxygen atom of umbelliferone and a hydrogen atom of the methyl group of alanine at the D2P8 position is 2.65 Å, and the Uele between these two atoms is approximately −2.32 kcal/mol, suggesting that this is a rather weak hydrogen bond. This suggests that alanine at D2P8 is adjacent to umbelliferone and is likely to interact. As a result of predicting amino acid positions that can interact with DMAPP and umbelliferone, 19 amino acid positions were found. Among them, the positions where the amino acid differs between the amino acid sequences of PsPT1 and PsPT2 are the D2P8 position and the D4P12 position (Supplementary Figs S1, S9B, and S10A). Furthermore, superimposition of the 3D models of PsPT1 and PsPT2 suggested that all amino acid residues close to umbelliferone and/or DMAPP are similarly oriented between these enzymes (Supplementary Fig. S9C). This result supported that mismatch positions, i.e. D2P8 and D4P12, are important to explain the difference in the regiospecificity between these enzymes.

### Conservation of D2P8 function in Apiaceae plants

**Figure 5.**
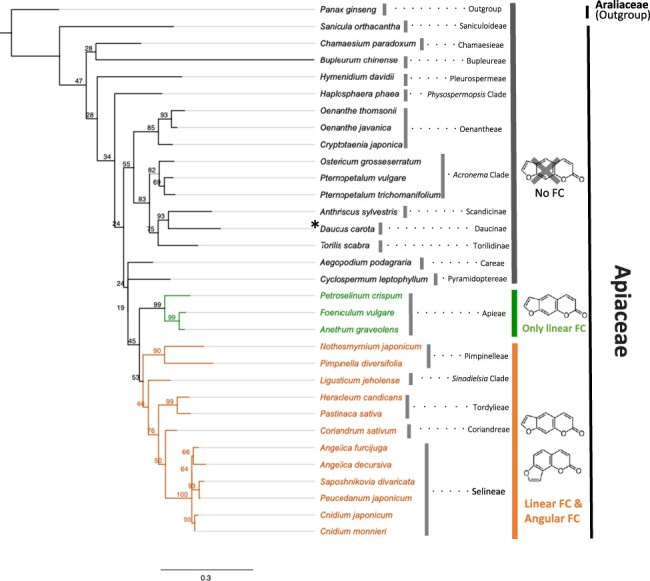
A phylogenetic tree of Apiaceae plant classification and distribution of furanocoumarins. A RAxML tree of Apiaceae ITS2 sequences was constructed with 1000 bootstrap tests based on a MAFFT alignment with the GAMMA JTT. The bar represents a nucleotide substitution rate per site of 0.30. Apiaceae plants containing no furanocoumarins, only linear furanocoumarins, and both linear and angular furanocoumarins are shown in gray, green, and orange, respectively. *Exceptionally, some reports have found furanocoumarins in *D. carota*, albeit at low levels. *Panax ginseng* in Araliaceae is used as an out-group.

We then investigated whether the regulation of the regiospecificity of the prenylation position by D2P8 is applicable to other umbelliferone dimethylallyltransferases (UDTs) in Apiaceae other than *P. sativa*. In Apiaceae, the ability to produce furanocoumarins is observed in a few limited species. Figure 5 shows the phylogeny of representative Apiaceae species based on internal transcribed spacer 2 (ITS2) sequences and the occurrence of linear and angular furanocoumarins. For example, the isolation of furanocoumarins has been reported in *Apium graveolens* and *Anethum foeniculum*, which contain only linear furanocoumarins, whereas *Heracleum sosnowskyi* and *Saposhnikovia divaricata* have been reported to contain both linear and angular furanocoumarins (Beier et al. 1983, Stavri and Gibbons 2005, Kim et al. 2008, Mishyna et al. 2015). The presence of furanocoumarins has not been reported in some species such as *Centella asiatica, Eryngium alpinum, Oenanthe javanica*, and *Oenanthe sinensis*. In *Daucus carota*, although we did not find the presence of *UDT* in its genome, the detection of an extremely small amount of furanocoumarin has been reported (Melough et al. 2017).

**Figure 6.**
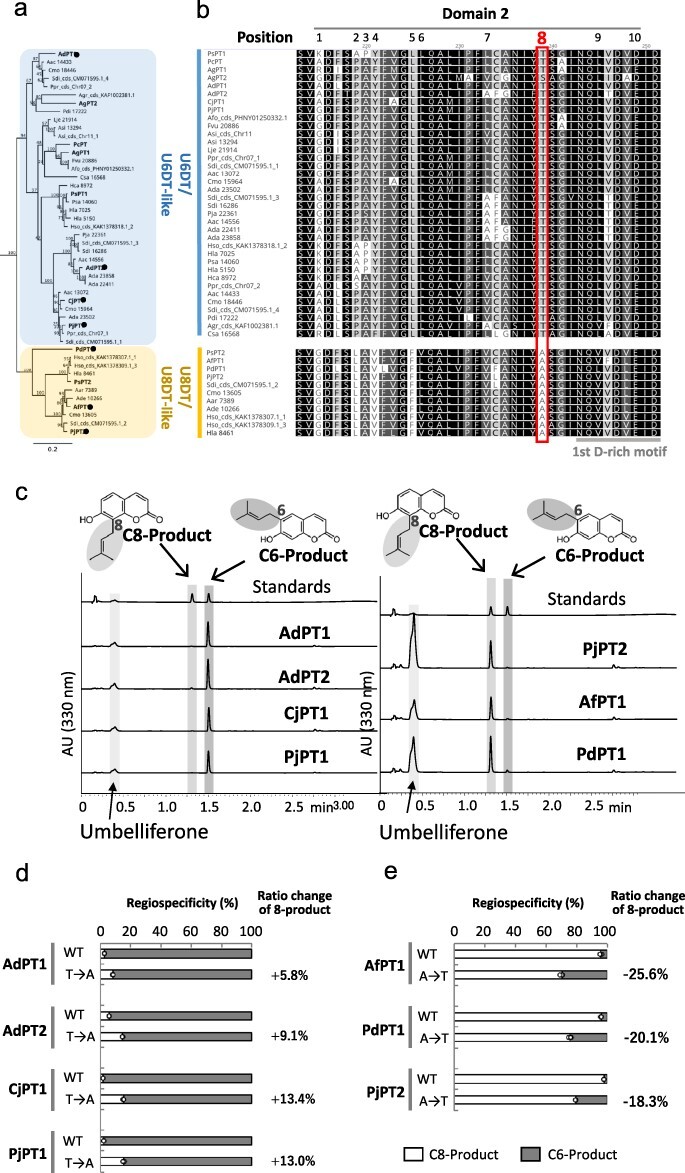
Isolation of new UDTs from various furanocoumarin-producing plants in Apiaceae. (a) The UDT clade of a phylogenetic tree of apiaceous PTs. Blue, the U6DT/U6DT-like group; yellow, the U8DT/U8DT-like clade. Bold: PTs with confirmed activity. Filled circles, Apiaceae PTs newly obtained in this experiment and used in mutagenesis (d, e). The scale bar represents an amino acid substitution rate of 0.2. The complete phylogenetic tree is shown in Supplementary Fig. S16. (b) Multiple alignment analysis of the domain 2 region of the apiaceous U6DT/U6DT-like group and the U8DT/U8DT-like group. (c) Enzyme reaction products of newly isolated PT from Apiaceae using umbelliferone and DMAPP as substrates. (d) Effect of the introduction of alanine (A) at position D2P8 of U6DTs on regiospecificity (*n* = 3). (e) Effect of introduction of threonine (T) at the D2P8 position of U8DTs on the regiospecificity (*n* = 3). For (d) and (e), white bar, prenylation product at the C8 position; gray bar, prenylation product at the C6 position.

As an example of new PT genes in Apiaceae, we cloned two *UDTs* from dill (*Anethum graveolens*), which were designated as *AgPT1* and *AgPT2*. The recombinant proteins of these membrane-bound PTs show the activity of U6DT (Supplementary Fig. S11). As it was difficult to prepare microsomes with high activity for AgPT2, detailed biochemical analysis using AgPT1 revealed that this enzyme has almost identical enzymatic properties to PsPT1, i.e. strict substrate specificity for both prenyl donor and acceptor substrates, apparent *K_m_* values, divalent cation preference, pH optimum and plastid localization (Supplementary Figs S12–S15). We also obtained new PT genes from different Apiaceae species capable of producing furanocoumarins: *PjPT1* and *PjPT2* from *Peucedanum japonicum, AdPT1* and *AdPT2* from *Angelica decursiva, AfPT1* from *Angelica furcijuga*, and *CjPT1* from *Cnidium japonicum* (Figs 5 and 6a, Supplementary Fig. S16). *PdPT1* was artificially synthesized from the sequence of transcriptome data of *Pimpinella diversifolia*. In the multiple alignment of these newly isolated PTs together with the known and predicted U6DT and U8DT of the Apiaceae, we found that the amino acids of the D2P8, namely four residues before the first D-rich motif, are highly conserved (Fig. 6b). The amino acids at the D2P8 position of the U6DT group are all alanine, whereas those of the U8DT members are all threonine except for AgPT2, which has low U6DT activity compared to AgPT1. In agreement with the prediction from these primary sequence analyses, the major prenylation activity of PjPT1, AdPT1, AdPT2, and CjPT1 was found to be the same as that of PsPT1 (U6DT), whereas the major PT activity of PjPT2, PdPT1, and AfPT1 was the same as that of PsPT2 (U8DT) (Fig. 6c).

We then introduced the alanine point mutation at position D2P8 of PjPT1, AdPT1, AdPT2, and CjPT1 in the U6DT group, and conversely the threonine point mutation at position D2P8 of PjPT2, PdPT1, and AfPT1 in the U8DT group. As a result, we found that these PTs, whether U6DT or U8DT, all showed the same regiospecific changes as PsPT1/2 (Fig. 6d and e). This shows that the role of D2P8 in regulating regiospecificity is highly conserved among Apiaceae UDT enzymes, emphasizing that this position is extremely important for the enzymatic function in all umbelliferone PTs of the Apiaceae family.

## Discussion

UbiA PTs are found in bacteria and humans and are involved in the biosynthesis of various metabolites. A representative compound synthesized by UbiA PTs is ubiquinone, an important quinone compound involved in the respiratory chain. Many members of this membrane-bound enzyme family catalyze the coupling reaction between an aromatic compound and the isoprenoid chain. Plants provide a large source of UbiA PT genes. While some PT members are involved in the biosynthesis of primary metabolites such as vitamin E, plastoquinone, chlorophyll, and ubiquinone, much more divergent members are found in secondary metabolism, the representative compounds being prenylated polyphenols. Indeed, plant PTs contribute significantly to the chemical diversity of secondary metabolites and also to their biological activities (Yazaki et al. 2009). From the point of view of biosynthetic studies, this family of enzymes occupies the pivotal position of combining two major biosynthetic pathways, i.e. the shikimate/polyketide pathway to provide aromatics and the mevalonate/nonmevalonate pathway to serve isoprenoids. Because of their importance, these UbiA PTs have been intensively studied since the first discovery of flavonoid-specific PTs (Sasaki et al. 2008, Munakata and Yazaki 2024). Molecular identification of PT sequences with different catalytic functions from different plant species has shown that plant PTs generally exhibit strict specificities for both substrates and products (Yang et al. 2023). This unique enzymatic feature has attracted much attention from plant biochemists, raising the question of what is the molecular mechanism that determines such specificities, while crystallographic studies have been hampered by the membrane-bound nature of this enzyme family. We therefore carried out a detailed biochemical analysis using domain-swapping and site-directed mutagenesis studies on two representative coumarin-specific PTs with different regiospecificities and high-sequence similarity, i.e. PsPT1 and PsPT2 from parsnip.

To express chimeric PT proteins, we chose the agroinfiltration method using *N. benthamiana* as a host organism to express plant PTs, which provides a higher success rate in obtaining plant membrane proteins compared to budding yeast, which is often used in previous studies (Sasaki et al. 2011, Yang et al. 2018). In fact, most chimera enzymes showed enzymatic activities. The higher amino acid identity between PsPT1 and PsPT2 suggests that their chimeric enzymes are more likely to retain activity by retaining their native higher order structure.

Plant PTs require Mg^2+^ for catalytic function, and two D-rich motifs, the first and second aspartate-rich motifs, are involved in substrate recognition via Mg^2+^. One of our findings for PsPT1 and PsPT2 in this study is that the domain 2 containing the first D-rich motif has the largest contribution to the regiospecificity of either C6- or C8-prenylation on the prenyl acceptor substrate umbelliferone. Site-directed mutagenesis studies revealed that the D2P8 position, located four residues before the first D-rich motif, has the most pronounced influence on regiospecificity, covering ∼60% of the influence of domain 2, i.e. threonine at this position drives the shift towards C6-prenylation, whereas alanine at this position drives the prenylation position towards C8.

Furthermore, our data on the detailed *K_m_* values of mutant enzymes suggest that the interaction with the acceptor substrate is the main regulator of the regiospecificity of PTs. It should be emphasized that the D2P8 position was identified by the 3D modeling studies independently of the biochemical experiments. In agreement with the biochemical results, this *in silico* analysis proposed an interaction between D2P8 and the prenyl acceptor substrate. For PsPT1, a hydrogen bond-based interaction was proposed between the threonine at D2P8 and the prenyl acceptor. However, this position may have an additional role, as substitution of valine for threonine at D2P8 resulted in only a small change in the regiospecificity of Ps21222. In a bacterial PT, it has recently been reported that the amino acid at position 7 upstream of the first D-rich motif is relevant for prenyl acceptor substrate specificity (Yang et al. 2023). The N-terminal region of the first D-rich motif may be involved in the interaction with the prenyl acceptor substrate of broad UbiA PT members in general.

This study also suggests previously unknown catalytic mechanisms that regulate regiospecificity. In PsPT2, we observed that the introduction of the mutation at D2P8 affected the apparent *K_m_* values not only for the prenyl acceptor but also for the prenyl donor, while our 3D modeling analysis did not give a clear insight to reveal this effect. In our mutagenesis experiment, the second important position in the regiospecificity was found to be D4P10 in domain 4. It should be noted that D4P10 is just outside the second D-rich motif towards the N-terminal flanking region, and the amino acid mutation of D4P10 and D2P8 positions showed an additive effect on the regiospecificity. In addition to D2P8 and D4P10, we also found residues affecting regiospecificity, e.g. D2P4, D2P7, and D4P12, with the effect of D2P4 being additive to that of D2P8. However, these five residues are distant from each other in 3D models of PsPT1 and PsPT2 (Fig. 4a, Supplementary Fig. S10A). In particular, D2P4 is opposite to the catalytic pocket. Therefore, direct interactions between them are unlikely and each of these residues may influence regiospecificity in its own way.

Apiaceae is a rich source of PT genes for the umbelliferone substrate. While representative U6DT and U8DT are shown in Fig. 6a, there are many more candidate genes encoding PT and PT-like polypeptides, and we have constructed a thorough phylogenetic tree using all candidate PT genes in Apiaceae (Supplementary Fig. S16). In Apiaceae, some plant species produce only linear furanocoumarins, such as parsley, and some plant species produce both linear and angular furanocoumarins, such as parsnip. In this study, we comprehensively collected UDT sequences in Apiaceae (Figs 5 and 6a), and the multiple alignment of their amino acids was observed. We then found that the amino acids at the D2P8 position of U6DT are all threonine except for AgPT2, while the amino acids at the D2P8 position of U8DT are all alanine, revealing the high amino acid conservation at this position, reflecting the regiospecificity of either U6DT or U8DT (Fig. 6b and c). These results strongly suggest that D2P8 is a common key control position for the regulation of regiospecificity in apiaceous UDTs. For the formation of furanocoumarins, *p*-coumaroyl-CoA 2ʹ-hydroxylase and prenyl chain cyclase are other key steps as the upstream and downstream steps of the prenylation reaction. A genome-based study of the molecular evolution of these three enzymes has recently been published (Huang et al. 2024), which speculates on the reason why only a fraction of plants in the Apiaceae synthesize furanocoumarin derivatives.

Plant–insect interactions have long been a topic of research, and coevolution of genes between insects and plants has often been reported (Sperling and Harrison 1994). Recently, it has been recognized that plant PTs have evolved through convergent evolution within the plant family, similar to P450 enzymes (Villard et al. 2021, Munakata et al. 2021, Munakata and Yazaki 2024). This regiospecificity of coumarin-specific PTs plays an important role in the biological stress tolerance of Apiaceae. Linear furanocoumarins show toxicity to some *Papilio* species, while other herbivores can degrade these metabolites, whereas angular furanocoumarins are more resistant to degradation and act as a stronger chemical barrier against specialist insects. There are apiaceous plants that produce either only linear furanocoumarins or both linear and angular furanocoumarins, but no plants are known to contain only angular furanocoumarins. This provides evidence that the biosynthetic pathway for linear furanocoumarins was developed first and that the angular derivatives were generated later (Berenbaum and Feeny 1981, Schuler et al. 2003). In the biosynthesis of both types of furanocoumarins, the fate of the compounds is determined by the regiospecificity of the PTs for umbelliferone, i.e. the former is formed by U6DT and the latter by U8DT. It is therefore hypothesized that U6DT was first generated from VTE2-1, which is responsible for tocopherol biosynthesis, and that the new U8DT has subsequently evolved as a neofunctionalization of U6DT in Apiaceae (Munakata et al. 2020). Our biochemical results may help to elucidate this molecular evolutionary event. When we exchanged domain 2 between PsPT1 and PsPT2, PsPT2 changed its regiospecificity more than PsPT1. The same result was obtained with a point mutation at D2P8. This difference in plasticity to mutagenesis between PsPT1 and PsPT2 suggests the possibility that C8-specificity was established by the acquisition of multiple residues based on the catalytic mechanism of U6DT. In other words, PsPT1 requires multiple residues to increase its C8 specificity, whereas PsPT2 can easily decrease its C8 specificity by a single mutation. In addition, the introduction of the PsPT2-like residue alanine at D2P8 had a greater effect on the regiospecificity of Ps21222 than PsPT1, suggesting that U8DTs have arisen by mutation(s) outside domain 2 as well as D2P8. Further phylogenetic and enzymological analyses will clarify the molecular evolutionary relationship between U6DTs and U8DTs.

Plant PT serves as an attractive molecular tool for synthetic biology to produce valuable plant products in microorganisms (de Bruijn et al. 2020). High regiospecificity is advantageous for the specific production of a particular target compound. Such regiospecificity is also known for the flavonoid substrate PT (Wang et al. 2014, Liu et al. 2018, 2021). Using a flavonoid-specific PT, a potential drug from *Epimedium* species, icaritin (a prenylated flavone), was produced in yeast and *Escherichia coli* (Wang et al. 2021). In our mutagenesis experiment, the introduction of cysteine at D2P8 increased the regiospecificity of Ps21222 and PsPT2, demonstrating the potential of this position in the engineering of plant PTs. However, there are still many aspects to be elucidated at the molecular level. The combination of biochemical analysis and 3D modeling studies using different pairs with different properties will provide a powerful tool to elucidate the function of plant PT in the future, which will contribute to the strategic production of target compounds in synthetic biology.

## Materials and Methods

### Plant materials

Leaves of *P. japonicum* were collected at the Yamashina Botanical Research Institute (Nippon Shinyaku Co., Ltd). After collection, *P. japonicum* leaves were immediately stored in a refrigerator at −80°C. *Angelica decursiva*, *A. furcijuga*, and *C. japonicum* plants were purchased commercially in Japan and grown in an indoor culture room at 25°C. *Nicotiana benthamiana* seeds were buried in culture soil and grown in a culture room at a temperature of 25°C.

### Chemical reagents

The prenyl acceptor substrate, umbelliferone, and the prenyl donor substrate, DMAPP, were purchased from Sigma Aldrich (https://www.sigmaaldrich.com/) and TargetMol (https://www.targetmol.com/). Osthenol used as a standard was purchased from ChemFaces (Wuhan, China). A standard sample of demethylsuberosin was purchased from Topharman (Shanghai, China).

### Domain substitution of PsPT1 and PsPT2

The cDNAs of *PsPT1* and *PsPT2* were recloned for this study (see below). We selected four shuffling regions where the amino acid sequences of PsPT1 and PsPT2 are identical (Supplementary Fig. S1). Each PT sequence was divided into five domains by the shuffling regions (domain 1, domain 2, domain 3, domain 4, and domain 5). The PCR reactions (PrimeSTAR Max Premix, Takara) were performed using primers (Supplementary Tables S3 and S4), and 30 different domain-substituted chimeric enzymes were constructed in the pENTR2B (Thermo Fisher Scientific, Waltham, MA, USA) vector using In-Fusion HD Cloning Kit (Takara, Kusatsu, Japan). The chimeric gene was then inserted into the pGWB502 vector (Nakagawa et al. 2007) using LR Clonase™ II enzyme (Thermo Fisher Scientific).

### Site-directed mutagenesis of enzymes

All point mutant enzymes were constructed using the KOD-Plus mutagenesis kit (Toyobo, Osaka, Japan). The primer designs are shown in Supplementary Table S5. The pENTR2B plasmid containing *PT* was used as the reaction substrate. After constructing the point mutation *PTs* on the pENTR2B plasmid, we introduced them into the pGWB502 vector in the same way as the domain replacement chimera enzyme.

### Construction of transcriptome data

All RNA-seq data for Apiaceae plants were obtained from the National Center for Biotechnology Information (NCBI) (https://www.ncbi.nlm.nih.gov/) (Supplementary Table S6). We selected a subset of Apiaceae plants based on the work of Wen et al. (2020). RNA-seq FASTQ files downloaded from the NCBI were trimmed using BBduk and assembled using SPAdes and Geneious Prime® 2023.2.1 (Dotmatics). Candidate PT contigs were narrowed down using the amino acid sequences of PsPT1 and PsPT2 as entries using NCBI-blast-2.12.0. Among contigs with coding sequences (CDSs) greater than 1000 bp, CDSs of contigs in which the presence of two D-rich motifs was confirmed were selected as candidate PT genes.

### Isolation of PT genes and construction of plant expression plasmids

For this study, cDNAs of *PsPT1* and *PsPT2* were recloned from parsnip leaves (cv. Demi-long de Guernesey) and roots (cv. Hollow Crown), respectively. For the cloning of *PsPT1*, leaves were ground with liquid nitrogen in mortar and pestle, and total RNA was extracted using the E.Z.N.A.® SP Plant RNA kit (Omega Bio-tek), and further cDNA synthesis was performed using the High Capacity RNA-to-cDNA Kit (Thermo Fisher Scientific). Cloning PCR was performed using PrimeSTAR Max DNA polymerase (Takara) and the primer pair PsPT1_Fw and PsPT1_Rv (Supplementary Table S7), which was cloned into the vector pCR™8/GW/TOPO™ by TOPO TA cloning for sequencing. The resulting sequence was identical to that registered with the NCBI as KM017083. For the cloning of *PsPT2*, roots were ground in a mortar and pestle using liquid nitrogen. Total RNA was extracted from the root powder using the RNeasy Plant Mini Kit (Qiagen, Hilden, Germany), and cDNA synthesis was performed using Rever Tra Ace (Toyobo). The complete CDS of *PsPT2* was amplified by PCR using the resulting cDNA pool as template, PrimeSTAR Max DNA polymerase, and the primer pair PsPT2_Fw and PsPT2_Rv (Supplementary Table S7). The PCR product was introduced into pGEM®-T-easy (Promega, Madison, WI, USA) for sequencing. The resulting sequence has a 4-bp mismatch with the reported *PsPT2* (KM017084.1) (Munakata et al. 2016), so we named the new sequence *PsPT2b*, which is referred to as *PsPT2* in this article.

All other *PTs* were isolated using the following protocols. RNA extracted from *A. graveolens* seedlings and leaves of the other apiaceous species using the RNeasy Plant Mini Kit (Qiagen) was treated with DNase (Ambion DNA-free DNase Treatment and Removal, Thermo Fisher Scientific) and reverse transcribed to cDNA (SuperScript IV Reverse Transcriptase, Thermo Fisher Scientific). Synthesized cDNAs are stored at −30°C, diluted, and used as templates for PCR reactions. Primers were designed based on the candidate *PT* sequences selected from RNA-seq data (Supplementary Table S7). Cloning PCR was performed using KOD FX Neo or PrimeSTAR Max. After purification, the PCR product was introduced into pGEM®-T Easy Vector Systems. The sequences of the extracted plasmids were confirmed by DNA sequencing. The obtained PT genes were transferred into the pENTR2B vector through In-Fusion reaction and finally introduced into the pGWB502 vector. For *AgPT1* and *AgPT2*, the CDSs were introduced into the pRI101-AN vector (Takara) by digestion and ligation.

### Expression of PT proteins in *N. benthamiana* and simplified production of crude enzymes containing membrane proteins

Plasmids harboring *PTs* were individually introduced into *A. tumefaciens* LBA4404 strain. *A. tumefaciens* LBA4404 transformants were added to Yeast Extract Beef extract (YEB) liquid culture medium, cultured to Optical Density at 600 nm (OD_600_) 0.9–1.5, centrifuged, washed, prepared with MilliQ, and thoroughly mixed with *Agrobacterium tumefaciens* C58C1 strain carrying the plasmid pBIN61-*P19* (Voinnet et al. 2003). In the suspension, the OD_600_ of the transformants with a *PT* and *P19* were adjusted to 0.2 and 0.4, respectively. The suspension was infiltrated into leaves of *N. benthamiana*. We selected *N. benthamiana* plants that were >6 weeks old and infiltrated *Agrobacterium* suspension into their healthy leaves (one leaf for expression of one construct). Four days after infiltration, the infected leaves were collected and frozen by liquid nitrogen. Each leaf was carefully ground to powder using a mortar and a pestle. The powder was transferred to 1.6 ml of 100 mM KPi buffer (10 mM dithiothreitol, pH 7.0) and homogenized by grinding with another mortar on ice. After centrifugation of the homogenate (10 000 × *g*, 4°C, 5 min), we collected the supernatant and centrifuged again (20 400 × *g*, 4°C, 20 min). After discarding the supernatant, we added 600 µl of 100 mM Tris-HCl solution (1 mM dithiothreitol, pH 8.0) to the pellet and resuspended to prepare crude enzymes.

### Preparation of microsomes using ultracentrifugation

The cell-free extract was subjected to ultracentrifugation (BECKMAN COULTER, 10 000 × *g*, 4°C, 1 h) to obtain crude enzymes. The microsomal fraction was resuspended in 1 ml of 100 mM Tris-HCl (1 mM dithiothreitol, pH 8.0) and used as crude enzymes. For kinetic studies, the microsomes were diluted 100-fold. The detailed protocol has been described in Munakata et al. (2016).

### *In vitro* PT assay

Standard UDT reaction mixtures (200 µl) containing crude enzymes (∼2 µg/reaction for the simplified preparation and ∼100 µg/reaction for the conventional preparation using ultracentrifugation), 200 µM DMAPP, 200 µM umbelliferone, and 10 mM MgCl_2_ were incubated at 28°C for 20 h. After incubation, the reaction products are extracted. We added 100 μl of 3 M hydrochloric acid to the incubation mixture to stop the reaction. Then 300 µl of ethyl acetate was added, shaken thoroughly, and centrifuged (4400 × *g*, RT, 5 min). A portion (200 µl) of the ethyl acetate fraction was collected and evaporated to dryness by vacuum centrifugation. We added 100 µl of methanol to completely dissolve the precipitate as enzyme reaction products for analysis.

### HPLC analysis of enzymatic reaction products

Enzyme reaction products were detected and quantified using a NEXERA UHPLC system (Shimadzu, Kyoto, Japan) equipped with a photodiode array (SPD-M40, Shimadzu). The chromatographic column was a C18 reverse phase column (ACQUITY UPLC BEH C18 Column, 130 Å, 1.7 µm, 2.1 mm × 50 mm; Waters, Milford, MA, USA). Enzyme products were separated using a gradient of solvent A [water with 0.1% (v/v) formic acid] and solvent B [acetonitrile with 0.1% (v/v) formic acid] consisting of a gradient of 20%–95% solvent B for 2.5 min, held for 1 min, returned to 20% solvent B for 0.1 min, held for 3.5 min. The flow rate was adjusted to 1.0 ml/min. Enzymatic products were quantified at 330 nm using standard samples of demethylsuberosin and osthenol. The molar ratio of C6-prenylated and C8-prenylated products was calculated from their peak area in HPLC using standard curves, and the ratio change of C8-prenylated product was calculated by [% mutant C8-position product] − [% wt C8-position product] (Supplementary Fig. S17).

### 3D modeling and simulation

The polypeptide sequences of PsPT1 and PsPT2 were submitted to the Alphafold2 program (Jumper et al. 2021) and their 3D structures were generated. Docking studies of DMAPP, unbelliferone, and Mg^2+^ as ligands and PTs were performed using the ASEDock program (MOLSIS Inc., Japan) (Goto and Kataoka 2008) on MOE 2020.09 (Chemical Computing Group ULC, Montreal, Canada). The energies of the bound structures were expressed as Udock (kcal/mol), which was calculated as follows:


$${\mathrm{Udock = Uele + Uvdw + Ustrain}}{\mathrm{.}}$$

Here, Uele, Uvdw, and Ustrain are Coulombic, van der Waals, and generalized Born/solvent accessible solvation interactions, respectively. The MMFF94x force field has been used to calculate each energy term. It is generally accepted that the lower the Udock value is, the more stable the complex structure is formed. CueMol2 (http://www.cuemol.org/ja/index.php?cuemol2) was also used to visualize 3D models.

### Phylogenetic tree

For the Apiaceae family classification tree, we selected plant species of the Apiaceae family with reference to the paper by Wen et al. (2021). The ITS2 sequences of each plant species were obtained from the NCBI (https://www.ncbi.nlm.nih.gov/) (Supplementary Table S8, Supplementary Fig. S18). Multiple Alignment using Fast Fourier Transform (MAFFT) (https://mafft.cbrc.jp/alignment/server/index.html) was used to align PT sequences. RAxML (https://cme.h-its.org/exelixis/web/software/raxml/) was used to construct the phylogenetic tree (GAMMA JTT, bootstrap replicates 1000). *PT* sequences were collected from public genome data (Supplementary Table S9) using blastn with known *PT* members as queries. Transcriptome data were also used to collect *PT* sequences (Supplementary Fig. S6). The selected candidate PTs of Apiaceae plants and previously reported PTs were subjected to phylogenetic tree analysis together (Supplementary Table S10, Supplementary Fig. S19). A multiple alignment and a phylogenetic tree were constructed as described for ITS2.

### Statistics and reproducibility

Enzymatic reactions were performed in triplicate. Apparent *K_m_* values were calculated by nonlinear least squares using Sigmaplot 14.5.

## Supplementary Material

pcae134_Supp

## Data Availability

The nucleotide sequences of *PsPT2b, AdPT1, AdPT2, CjPT1, PjPT1, PjPT2, AfPT1, AgPT1, AgPT2*, and *PdPT1* are available in the NCBI under the accession numbers LC847665, LC847666, LC847667, LC847668, LC847669, LC847670, LC847671, LC847672, LC847673, and LC847674, respectively.
