# Supplementary material for: Catalytic mechanism underlying the regiospecificity of coumarin-substrate transmembrane prenyltransferases in Apiaceae
Source: Plant Cell Physiol. 2024 Nov 22;66(1):1–14. doi: 10.1093/pcp/pcae134 (PMC11775389; doi:10.1093/pcp/pcae134)
Supplement: pcae134_Supp [file pcae134_supp.zip › suppl_data/pcp-2024-e-00219-File009.pdf]

Supplementary data

Han et al.

Materials and Methods

Microscopic observation of the GFP fusion protein in Suppl. Fig. S15.

The first 150 bp regions of the CDSs of *AgPT1* and *AgPT2*, which contain the sequences encoding their predicted transit peptide (TP), were amplified by PCR using the pGEM®-T-easy vector containing the complete CDSs of *AgPT1* and *AgPT2* as a template, KOD FX Neo polymerase (TOYOBO), and the primer pairs for *AgPT1* (Fw, 5’-GAATTCATGACTCAAACATTTATGCAT-3’; Rv, 5’-GGATCCAGAAACCACCCTGAAAGG-3’) and *AgPT2* (Fw, 5’-GAATTCATGCCTCAAACATTTATGCAT-3’; Rv, 5’-GGATCCAGAAACCACCCTGGAAGG -3’). The amplicons (*AgPT1-TP* and *AgPT2-TP*) were introduced into the pGEM®-T-easy vector by TA cloning. Subsequently, *AgPT1-TP* and *AgPT2-TP* were fused to the 5’-terminus of *GFP* introduced at the BamHI and NotI sites in the pBluescript SK(-) vector by double digestion with EcoRI and BamHI and ligation. The *AgPT1-TP-GFP* and *AgPT2-TP-GFP* were introduced into pENTR1A (Thermo Fisher Scientific) by double digestion with EcoRI and NotI and ligation using T4 DNA ligase (Promega) and finally into the pEAQ-HT-DEST1 vector by LR reaction using Gateway™ LR Clonase™ II Enzyme mix (Thermo Fisher Scientific). This vector allows for high production of recombinant proteins due to the cowpea mosaic virus hypertranslational expression system (Peyret et Lomonossoff, 2013). The resulting plasmids pEAQ-HT-DEST1-*AgPT1-TP-GFP* and pEAQ-HT-DEST1-*AgPT2-TP-GFP* were transiently expressed in *Nicotiana benthamiana* leaves by agroinfiltration as described (Munakata et al., 2016). Microscopy was performed with Leica AF6000LX.

References

Peyret, H., Lomonossoff, G.P. (2013) The pEAQ vector series: the easy and quick way to produce recombinant proteins in plants, *Plant Mol. Biol.*, 83: 51-58.

Munakata, R., Olry, A., Karamat, F., Courdavault, V., Sugiyama, A., Date, Y., et al. (2016) Molecular evolution of parsnip (*Pastinaca sativa*) membrane-bound prenyltransferases for linear and/or angular furanocoumarin biosynthesis. *New Phytol.*, 211: 332-344.

Table S1. Amino acid identities between two UbiA PTs with different reaction specificities.

Rank	PT pair		Difference in reaction specificity	AA identity
1	PsPT1	PsPT2	Regio	70.9
2	GmC4DT	GmIDT1	Acceptor	68.9
3	PcPT	PsPT2	Regio	68.9
4	SfN8DT-2	SfG6DT	Accepor/Regio	68.3
5	GmIDT1	GmIDT2	Regio	67.2
6	SfG6DT	SfiLDT	Acceptor	67.1
7	GmIDT2	GmG2DT	Acceptor	67.1
8	GmC4DT	GmIDT2	Acceptor	66.4
9	SfFPT	SfG6DT	Acceptor/Regio	66.3
10	GuA6DT	GuILDT	Acceptor	66.2

The pairing of 60 PT sequences yielded 1,770 PT pairs. The pairs with different reaction specificities were selected and sorted according to their amino acid identities. The ten pairs with the highest amino acid identities are shown in the table.

Table S2. Results of docking simulation.

Entry	U_dock (kcal/mol)	DMAPP (C1)–Umb (C6) (U6DT activity)	DMAPP (C1)–Umb (C8) (U8DT activity)
**A. PsPT1 (U6DT)**			
1	-2636.49	7.23	8.66
2	-2475.20	6.00	8.37
3	-2334.91	6.91	8.99
4	-2310.53	4.66	6.74
5	-2289.47	7.24	6.68
**6**	**-2273.63**	**3.81**	**5.90**
7	-2271.67	5.88	6.19
8	-2256.81	6.26	6.65
9	-2256.19	4.71	5.80
10	-2225.48	3.95	6.28
**B. PsPT2 (U8DT)**			
1	-1951.12	9.87	10.35
2	-1909.26	3.31	4.02
**3**	**-1899.86**	**5.66**	**3.63**
4	-1885.96	4.74	3.83
5	-1860.52	5.85	3.83
6	-1818.14	5.07	3.73
7	-1786.76	3.29	4.80
8	-1766.67	5.30	3.55
9	-1763.92	4.35	3.95
10	-1757.79	3.45	4.28

The docking simulation was performed for PsPT1 (A) or PsPT2 (B), substrates, and Mg^{2+}. The resulting 3D models (entries) are listed by energetic stability, which is shown as U_dock energy (kcal/mol). For each model, the capacity to catalyze the U6DT reaction was evaluated by the distances between C1 of DMAPP and C6 of umbelliferone with the threshold set as 4Å. Similarly, the capacity to catalyze U8DT reaction was evaluated by the distances between C1 of DMAPP and C8 of umbelliferone with the threshold set as 4Å. Entry 6 and 3 were further analyzed in this study as suitable models for PsPT1 and PsPT2, respectively.

Table S3. Combinations of templates and primer pairs for domain swapping.

In-fusion product	PCR fragment	Template	Fw primer	Rv primer
Ps11111 (*PsPT1*)	1	*PsPT1*	PsPT1_NB_Fw	PsPT1_CX_Rv
Ps22222 (*PsPT2*)	1	*PsPT2*	PsPT2_NB_Fw	PsPT2_CX_Rv
Ps11112	1	*PsPT1*	PsPT1_NB_Fw	PsPT1_IV_Rv
	2	*PsPT2*	PsPT2_IV_Fw	PsPT2_CX_Rv
Ps11122	1	*PsPT1*	PsPT1_NB_Fw	PsPT1_III_Rv
	2	*PsPT2*	PsPT2_III_Fw	PsPT2_CX_Rv
Ps11222	1	*PsPT1*	PsPT1_NB_Fw	PsPT1_II_Rv
	2	*PsPT2*	PsPT2_II_Fw	PsPT2_CX_Rv
Ps12222	1	*PsPT1*	PsPT1_NB_Fw	PsPT1_I_Rv
	2	*PsPT2*	PsPT2_I_Fw	PsPT2_CX_Rv
Ps22221	1	*PsPT2*	PsPT2_NB_Fw	PsPT2_IV_Rv
	2	*PsPT1*	PsPT1_IV_Fw	PsPT1_CX_Rv
Ps22211	1	*PsPT2*	PsPT2_NB_Fw	PsPT2_III_Rv
	2	*PsPT1*	PsPT1_III_Fw	PsPT1_CX_Rv
Ps22111	1	*PsPT2*	PsPT2_NB_Fw	PsPT2_II_Rv
	2	*PsPT1*	PsPT1_II_Fw	PsPT1_CX_Rv
Ps21111	1	*PsPT2*	PsPT2_NB_Fw	PsPT2_I_Rv
	2	*PsPT1*	PsPT1_I_Fw	PsPT1_CX_Rv
Ps21112	1	*PsPT2*	PsPT2_NB_Fw	PsPT2_I_Rv
	2	*Ps11112*	PsPT1_I_Fw	PsPT2_CX_Rv
Ps22112	1	*PsPT2*	PsPT2_NB_Fw	PsPT2_II_Rv
	2	*Ps11112*	PsPT1_II_Fw	PsPT2_CX_Rv
Ps22212	1	*PsPT2*	PsPT2_NB_Fw	PsPT2_III_Rv
	2	*Ps11112*	PsPT1_III_Fw	PsPT2_CX_Rv
Ps21122	1	*PsPT2*	PsPT2_NB_Fw	PsPT2_I_Rv
	2	*Ps11122*	PsPT1_I_Fw	PsPT2_CX_Rv
Ps22122	1	*PsPT2*	PsPT2_NB_Fw	PsPT2_II_Rv
	2	*Ps11122*	PsPT1_II_Fw	PsPT2_CX_Rv
Ps21222	1	*PsPT2*	PsPT2_NB_Fw	PsPT2_I_Rv
	2	*Ps11222*	PsPT1_I_Fw	PsPT2_CX_Rv
Ps12221	1	*PsPT1*	PsPT1_NB_Fw	PsPT1_I_Rv
	2	*Ps22221*	PsPT2_I_Fw	PsPT1_CX_Rv
Ps11221	1	*PsPT1*	PsPT1_NB_Fw	PsPT1_II_Rv
	2	*Ps22221*	PsPT2_II_Fw	PsPT1_CX_Rv
Ps11121	1	*PsPT1*	PsPT1_NB_Fw	PsPT1_III_Rv
	2	*Ps22221*	PsPT2_III_Fw	PsPT1_CX_Rv
Ps12211	1	*PsPT1*	PsPT1_NB_Fw	PsPT1_I_Rv
	2	*Ps22211*	PsPT2_I_Fw	PsPT1_CX_Rv

Table S3. Combinations of templates and primer pairs for domain swapping.-*continued*

In-fusion product	PCR fragment	Template	Fw primer	Rv primer
Ps11211	1	*PsPT1*	PsPT1_NB_Fw	PsPT1_II_Rv
	2	*Ps22211*	PsPT2_II_Fw	PsPT1_CX_Rv
Ps12111	1	*PsPT1*	PsPT1_NB_Fw	PsPT1_I_Rv
	2	*Ps22111*	PsPT2_I_Fw	PsPT1_CX_Rv
Ps12112	1	*Ps12222*	PsPT1_NB_Fw	PsPT2_II_Rv
	2	*Ps11112*	PsPT1_II_Fw	PsPT2_CX_Rv
Ps12212	1	*Ps12222*	PsPT1_NB_Fw	PsPT2_III_Rv
	2	*Ps11112*	PsPT1_III_Fw	PsPT2_CX_Rv
Ps11212	1	*Ps11222*	PsPT1_NB_Fw	PsPT2_III_Rv
	2	*Ps11112*	PsPT1_III_Fw	PsPT2_CX_Rv
Ps12122	1	*Ps12222*	PsPT1_NB_Fw	PsPT2_II_Rv
	2	*Ps11122*	PsPT1_II_Fw	PsPT2_CX_Rv
Ps21221	1	*Ps21111*	PsPT2_NB_Fw	PsPT1_II_Rv
	2	*Ps22221*	PsPT2_II_Fw	PsPT1_CX_Rv
Ps21121	1	*Ps21111*	PsPT2_NB_Fw	PsPT1_III_Rv
	2	*Ps22221*	PsPT2_III_Fw	PsPT1_CX_Rv
Ps22121	1	*Ps22111*	PsPT2_NB_Fw	PsPT1_III_Rv
	2	*Ps22221*	PsPT2_III_Fw	PsPT1_CX_Rv
Ps21211	1	*Ps21111*	PsPT2_NB_Fw	PsPT1_II_Rv
	2	*Ps22211*	PsPT2_II_Fw	PsPT1_CX_Rv
Ps12121	1	*Ps12111*	PsPT1_NB_Fw	PsPT1_III_Rv
	2	*Ps12221*	PsPT2_III_Fw	PsPT1_CX_Rv
Ps21212	1	*Ps21111*	PsPT2_NB_Fw	PsPT1_II_Rv
	2	*Ps22212*	PsPT2_II_Fw	PsPT2_CX_Rv

Table S4. Primer sequences used for domain swapping.

Name	Sequence (5'->3')
PsPT1_NB_Fw	CCAATTCAGTCGACTGGATCATGGCTCAAACAATTATGCA
PsPT1_CX_Rv	GCTGGGTCTAGATATCTCGATCAGCGCATGAAATGAATTA
PsPT2_NB_Fw	CCAATTCAGTCGACTGGATCATGACTCAGACACTTATGCA
PsPT2_CX_Rv	GCTGGGTCTAGATATCTCGATCAGCGCATGAAATGAATCA
PsPT1_I_Fw	GTTTCTCTGCTGCCCTTAACTTC
PsPT1_I_Rv	GTTAAGGGCAGCAGAGAAACC
PsPT2_I_Fw	GTTTCTCTGCTGCCCTTAACCTC
PsPT2_I_Rv	GTTAAGGGCAGCAGAGAAACTGA
PsPT1_II_Fw	ATAGACAAAATAAACAAGCCTTACCTG
PsPT1_II_Rv	GGCTTGTTTATTTTGTCTATGTCCA
PsPT2_II_Fw	ATAGACAAAATAAACAAGCCCTATCTT
PsPT2_II_Rv	GGCTTGTTTATTTTGTCTATTTCCA
PsPT1_III_Fw	CTAATGGGACTCACGATCCA
PsPT1_III_Rv	TGGATCGTGAGTCCCATTAG
PsPT2_III_Fw	CTAATGGGACTCACGATCCAGCCTTCTG
PsPT2_III_Rv	TGGATCGTGAGTCCCATTAGACCA
PsPT1_IV_Fw	CAGTGTGAGATATGGCCAAGAG
PsPT1_IV_Rv	CTTGGCCATATCTCACACTGAA
PsPT2_IV_Fw	CAGTGTGAGATATGGCCAAGAGAA
PsPT2_IV_Rv	CTTGGCCATATCTCACACTGAATG

Table S5. Primer pairs used for point mutagenesis.

Name	Sequence (5'->3')
PsPT2-D2P1(K)_Fw	CGGTCTCCAAAGATTTCTCACTTGCAGTT
PsPT2-D2P1(K)_Rv	CGGTCTCGCTTTGACTGAGGTTAAGGGCAG
PsPT2-D2P2(A)_Fw	CGGTCTCCGCTGCAGTTTTTGTGGGATTC
PsPT2-D2P2(A)_Rv	TGGTCTCCGAGCTGAGAAATCTCCGACTGA
PsPT2-D2P3(P)_Fw	CGGTCTCGCCAGTTTTTGTGGGATTCGTA
PsPT2-D2P3(P)_Rv	TGGTCTCTATGGAAGTGAGAAATCTCCGAC
PsPT2-D2P4(Y)_Fw	CGGTCTCGTATTTTGTGGGATTCGTACAG
PsPT2-D2P4(Y)_Rv	CGGTCTCTAATATGCAAGTGAGAAATCTCC
PsPT2-D2P5(L)_Fw	CGGTCTCGTTAGTACAGGCATTGATCCCG
PsPT2-D2P5(L)_Rv	CGGTCTCTGTAATCCCACAAAAACTGCAAG
PsPT2-D2P6(L)_Fw	TGGTCTCCCTACAGGCATTGATCCCGTTT
PsPT2-D2P6(L)_Rv	CGGTCTCTGTAGGAATCCCACAAAAACTGC
PsPT2-D2P7(L)_Fw	CGGTCTCCCTTTGTGCGAACATTTATGCC
PsPT2-D2P7(L)_Rv	TGGTCTCTAAAGAAACGGGATCAATGCCTGT
PsPT2-D2P8(T)_Fw	CGGTCTCCACCTCAGGAATAAATCAAGTG
PsPT2-D2P8(T)_Rv	CGGTCTCTAGGTATAAATGTTCGCACAAAC
PsPT2-D2P9(L)_Fw	CGGTCTCCCTTGTTGATGTGGAAATAGAC
PsPT2-D2P9(L)_Rv	CGGTCTCTCAAGTTGATTTATTCCTGAGGC
PsPT2-D2P10(D)_Fw	CGGTCTCCGACATAGACAAAATTAACAA
PsPT2-D2P10(D)_Rv	CGGTCTCTTGTCCACATCAACCACTTGATT

Table S5. Primer pairs used for point mutagenesis. - *continued*

Name	Sequence (5'->3')
PsPT2-D4P1(A)_Fw	GCTGTCTACTATCACATCCAA
PsPT2-D4P1(A)_Rv	AGGCTGAATGGTGAGTCC
PsPT2-D4P2(F)_Fw	TTCTATCACATCCAAAACGTGC
PsPT2-D4P2(F)_Rv	GACAGAAGGCTGAATGGTGA
PsPT2-D4P3(A)_Fw	GCACTTGGTAGACCAATGGTTT
PsPT2-D4P3(A)_Rv	GTTTTGGATGTGATAGTAGACAG
PsPT2-D4P4(K)_Fw	AAACCAATGGTTTTGACAAAGC
PsPT2-D4P4(K)_Rv	ACCAAGCACGTTTTGGATG
PsPT2-D4P5(F)_Fw	TTTACAAAGCCAGTGGTCTTCG
PsPT2-D4P5(F)_Rv	AACCATTGGTCTACCAAGC
PsPT2-D4P6(S)_Fw	TCAAAGCCAGTGGTCTTCGC
PsPT2-D4P6(S)_Rv	CAAAACCATTGGTCTACC
PsPT2-D4P7(T)_Fw	ACAGTGGTCTTCGCTACCAG
PsPT2-D4P7(T)_Rv	CTTTGTCAAAACCATTGG
PsPT2-D4P8(A)_Fw	GCCTTCGCTACCAGTTTCATCA
PsPT2-D4P8(A)_Rv	CACTGGCTTTGTCAAAAC
PsPT2-D4P9(I)_Fw	ATTTTCATCAGTGTCTTTTCAGC
PsPT2-D4P9(I)_Rv	GGTAGCGAAGACCACTGGCT
PsPT2-D4P10(F)_Fw	TTCAGTGTCTTTTCAGCTGTTC
PsPT2-D4P10(F)_Rv	GAAACTGGTAGCGAAGAC
PsPT2-D4P11(A)_Fw	GCAGCTGTTCTTGCAATGATCAA
PsPT2-D4P11(A)_Rv	AAAGACACTGATGAAACTGG
PsPT2-D4P12(G)_Fw	GGAATGATCAAGGACTTACC
PsPT2-D4P12(G)_Rv	AAGAACAGCTGAAAAGACAC
PsPT2-D4P13(A)_Fw	GCGATCAAGGACTTACCTGATG
PsPT2-D4P13(A)_Rv	TGCAAGAACAGCTGAAAAGA
PsPT2-D4P14(V)_Fw	GTACCTGATGTTGAAGGAGACAG
PsPT2-D4P14(V)_Rv	GTCCTTGATCATTGCAA
PsPT2-D4P15(T)_Fw	ACAGCGTTGGGCAACCTAACATTC
PsPT2-D4P15(T)_Rv	GTCTCCTTCAACATCAGGTA
PsPT2-D4P16(F)_Fw	TTCGGCAACCTAACATTCAGTG
PsPT2-D4P16(F)_Rv	CGCTCTGTCTCCTTCAACAT
PsPT2-D4P17(R)_Fw	CGAACATTCAGTGTTAGATATGG
PsPT2-D4P17(R)_Rv	GTTGCCCAACGCTCTGTCTC

Table S5. Primer pairs used for point mutagenesis. - *continued*

Name	Sequence (5'->3')
Ps21222_D2P8_All_Rv	ATAGATGTTGGCACAAAGAA
Ps21222_D2P8(A)_Fw	GCCTCGGGGATAAATCAACTTGT
Ps21222_D2P8(V)_Fw	GTCTCGGGGATAAATCAACTTGT
Ps21222_D2P8(N)_Fw	AACTCGGGGATAAATCAACTTGT
Ps21222_D2P8(R)_Fw	AGATCGGGGATAAATCAACTTGT
Ps21222_D2P8(W)_Fw	TGGTCGGGGATAAATCAACTTGT
Ps21222_D2P8(F)_Fw	TTCTCGGGGATAAATCAACTTGT
Ps21222_D2P8(L)_Fw	TTATCGGGGATAAATCAACTTGT
Ps21222_D2P8(I)_Fw	ATTTCGGGGATAAATCAACTTGT
Ps21222_D2P8(M)_Fw	ATGTCGGGGATAAATCAACTTGT
Ps21222_D2P8(S)_Fw	TCTTCGGGGATAAATCAACTTGT
Ps21222_D2P8(P)_Fw	CCTTCGGGGATAAATCAACTTGT
Ps21222_D2P8(Y)_Fw	TATTCGGGGATAAATCAACTTGT
Ps21222_D2P8(H)_Fw	CATTCGGGGATAAATCAACTTGT
Ps21222_D2P8(Q)_Fw	CAATCGGGGATAAATCAACTTGT
Ps21222_D2P8(K)_Fw	AAGTCGGGGATAAATCAACTTGT
Ps21222_D2P8(D)_Fw	GATTCGGGGATAAATCAACTTGT
Ps21222_D2P8(E)_Fw	GAATCGGGGATAAATCAACTTGT
Ps21222_D2P8(C)_Fw	TGTTCGGGGATAAATCAACTTGT
Ps21222_D2P8(G)_Fw	GGTTCGGGGATAAATCAACTTGT
PsPT2-D2P8_All_Rv	ATAAATGTTCGCACAAACAA
PsPT2_D2P8(C)_Fw	TGTTCAGGAATAAATCAAGTGGT
PsPT2_D2P8(V)_Fw	GTCTCAGGAATAAATCAAGTGGT
PsPT2_D2P8(G)_Fw	GGTTCAGGAATAAATCAAGTGGT
PsPT2_D2P8(N)_Fw	AACTCAGGAATAAATCAAGTGGT
PsPT2_D2P8(R)_Fw	AGATCAGGAATAAATCAAGTGGT
PsPT2_D2P8(W)_Fw	TGGTCAGGAATAAATCAAGTGGT
PsPT2_D2P8(F)_Fw	TTCTCAGGAATAAATCAAGTGGT
PsPT2_D2P8(L)_Fw	TTATCAGGAATAAATCAAGTGGT
PsPT2_D2P8(I)_Fw	ATTTCAGGAATAAATCAAGTGGT
PsPT2_D2P8(M)_Fw	ATGTCAGGAATAAATCAAGTGGT
PsPT2_D2P8(S)_Fw	TCTTCAGGAATAAATCAAGTGGT
PsPT2_D2P8(P)_Fw	CCTTCAGGAATAAATCAAGTGGT
PsPT2_D2P8(Y)_Fw	TATTCAGGAATAAATCAAGTGGT
PsPT2_D2P8(H)_Fw	CATTCAGGAATAAATCAAGTGGT
PsPT2_D2P8(Q)_Fw	CAATCAGGAATAAATCAAGTGGT
PsPT2_D2P8(K)_Fw	AAGTCAGGAATAAATCAAGTGGT
PsPT2_D2P8(D)_Fw	GATTCAGGAATAAATCAAGTGGT
PsPT2_D2P8(E)_Fw	GAATCAGGAATAAATCAAGTGGT

Table S5. Primer pairs used for point mutagenesis. - *continued*

Name	Sequence (5'->3')
PjPT2-D2P8_All_Rv	ATAAATGTTCGCAAGAA
PjPT2_D2P8(C)_Fw	TGTTCCGGAATAAATCAAGTGGT
PjPT2_D2P8(T)_Fw	ACCTCCGGAATAAATCAAGTGGT
AfPT1_D2P8_All_Rv	ATAAATGTTCGCACAAACA
AfPT1_D2P8(T)_Fw	ACCTCCGGAATAAATCAAGTGT
PjPT1-D2P8_All_Rv	ATAGATGTTCGCACAAAGAA
PjPT1_D2P8(C)_Fw	TGTTCGGGGATTAATCAACTGGT
PjPT1_D2P8(A)_Fw	GCCTCGGGGATTAATCAACTGGT
AdPT1_D2P8_All_Rv	GTAGATGTTCGCACAAACG
AdPT1_D2P8(A)_Fw	GCCTCCGGGATAAATCAACTGG
AdPT1_D2P8(C)_Fw	TGTTCCGGGATAAATCAACTGG
AdPT2_D2P8_All_Rv	AAAGATGTTCCCAAAAGCAA
AdPT2_D2P8(A)_Fw	GCCTCGGGGATAAATCAACTGAC
AdPT2_D2P8(C)_Fw	TGTTCGGGGATAAATCAACTGAC
PdPT1_D2P8_All_Rv	ATAGATGTTCGCAAAAACAAA
PdPT1_D2P8(T)_Fw	ACCTCGGGAATAAATCAATTGGT
PdPT1_D2P8(C)_Fw	TGTTCGGGAATAAATCAATTGGT

Table S6. Transcriptome data used in phylogenetic analysis.

Plant species	NCBI accession ID
Aegopodium podagraria	SRR8863753
Angelica acutiloba	SRR8863734
Angelica archangelica	ERR2040639
Angelica dahurica	SRR7876668
Angelica decursiva	SRR8863756
Angelica sinensis	SRR10524264
Anthriscus sylvestris	SRR8863738
Bupleurum chinense	SRR8863755
Centella asiatica	SRR12640679
Chamaesium paradoxum	SRR8863751
Cnidium monnieri	SRR8863740
Coriandrum sativum	SRR8863732
Cryptotaenia japonica	SRR8863737
Cyclospermum leptophyllum	SRR8863739
Ferula assa-foetida	SRR7366944
Foeniculum vulgare	SRR8863742
Glehnia littoralis	SRR5579881
Haplosphaera phaea	SRR8863746
Heracleum lanatum	ERR2040638
Heracleum candicans	SRR8863733
Heracleum sphondylium	SRR11487699
Hydrocotyle batrachium	SRR12009666
Hymenidium davidii	SRR8863750
Ligusticum jeholense	SRR8863748
Nothosmyrnium japonicum	SRR8863757
Oenanthe javanica	SRR8863744
Oenanthe thomsonii	SRR8863743
Pastinaca sativa	SRR8863749
Peucedanum japonicum	SRR8863747
Pimpinella diversifolia	SRR8863741
Pternopetalum trichomanifolium	SRR8863745
Pternopetalum vulgare	SRR8863736
Sanicula orthacantha	SRR8863735
Saposhnikovia divaricate	SRR8863754
Thapsia garganica	SRR343120
Torilis scabra	SRR8863758

Table S7. Primer pairs used for cloning and subcloning of native UbiA PTs.

Name	Sequence (5'->3')
AdPT1_Fw	ATGGCGGGGAGAGCTTCATA
AdPT1_Rv	AAGATGAAGAAACTGGCGCA
AdPT2_Fw	CATTGAAGTAACGCGGGCC
AdPT2_Rv	TCTGCTTGTCCCAACACCTT
AfPT1_Fw	GGGGGACGATAATAAACCAGC
AfPT1_Rv	TACCTCGGACGCCTATCTCT
AgPT1_Fw	CATATGACTCAAACATTTATGCATTCA
AgPT1_Rv	GGATCCTCAGCGCATGAAATGAATTAGC
AgPT2_Fw	CATATGCCTCAAACATTTATGCATTC
AgPT2_Rv	GAATTCTCAGCGCATAAAATGAATCAGG
CjPT1_Fw	CCACAGTCATCATAAACGAACC
CjPT1_Rv	CTGCATGTCCTCTGTTTGCTG
PjPT1_Fw	CAGACTCCACCCACCACAG
PjPT1_Rv	AAGAAGAGAAAGCCACGGGT
PjPT2_Fw	GGAGAGACTCCACAAGGCTA
PjPT2_Rv	CCTCGGAAACCTATCTCTGCT
PsPT1_Fw	AGCCGAAAAGCAGAGCCTAT
PsPT1_Rv	ATCCACAGTGGGTGACAACA
PsPT2_Fw	CCCACTGTGGTCATAAACGA
PsPT2_Rv	CCTTGGAAACCTATATCTGG
AdPT1_InFw	CCAATTCAGTCGACTGGATCATGTCTCAGACATTTATGC
AdPT1_InRv	GCTGGGTCTAGATATCTCGATCAGCGCATGAAATGAATCA
AdPT2_InFw	CCAATTCAGTCGACTGGATCATGACTCAGACACTTATGCA
AdPT2_InRv	GCTGGGTCTAGATATCTCGATCAGCGCATGAAATGAATTA
AfPT1_InFw	CCAATTCAGTCGACTGGATCATGACTCAAGCATTTATG
AfPT1_InRv	GCTGGGTCTAGATATCTCGATCAGCGAATGAAATGAATCA
CjPT1_InFw	CCAATTCAGTCGACTGGATCATGACTCAGACACTTATGCA
CjPT1_InRv	GCTGGGTCTAGATATCTCGATCAGCGCATGAAATGAATTA
PdPT1_InFw	CCAATTCAGTCGACTGGATCATGGCTCAGGCGTCTATG
PdPT1_InRv	GCTGGGTCTAGATATCTCGATTAGCGCATGAAATGTATC
PjPT1_InFw	CCAATTCAGTCGACTGGATCATGACTCAGACTCTTATGCA
PjPT1_InRv	GCTGGGTCTAGATATCTCGATCAGCGCATGAAATGAATTA
PjPT2_InFw	CCAATTCAGTCGACTGGATCATGACTCAAGCATCTATG
PjPT2_InRv	GCTGGGTCTAGATATCTCGATCAGCGAATGAAATGAATCAG

Table S8. Accession IDs of ITS2 sequences used in this study.

Plant species	Accession ID
Aegopodium podagraria	JF807549.1
Anethum graveolens	OQ165215.1
Angelica decursiva	MF589393.1
Angelica furcijuga	DQ278164.1
Anthriscus sylvestris	KJ956575.1
Bupleurum chinense	KJ716418.1
Chamaesium paradoxum	EU236161.1
Cnidium japonicum	MK036578.1
Cnidium monnieri	AY330507.1
Coriandrum sativum	JF807555.1
Cryptotaenia japonica	MG731061.1
Cyclospermum leptophyllum	KY968851.1
Daucus carota	AY065313.1
Foeniculum vulgare	HQ377208.1
Haplosphaera phaea	AY330514.1
Heracleum candicans	OP090053.1
Hymenidium davidii	FJ483479.1
Ligusticum jeholense	OL473115.1
Nothosmyrnium japonicum	DQ516367.1
Oenanthe javanica	MH711460.1
Oenanthe thomsonii	EU236186.1
Ostericum grosseserratum	MT735398.1
Panax ginseng	JX996141.1
Pastinaca sativa	MT254214.1
Petroselinum crispum	U78447.1
Peucedanum japonicum	AH012693.2
Pimpinella diversifolia	MF785522.1
Pternopetalum trichomanifolium	KP940747.1
Pternopetalum vulgare	AY038218.1
Sanicula orthacantha	AF031963.1
Saposhnikovia divaricate	OL473103.1
Torilis scabra	AF077120.1

Table S9. Genome data used in phylogenetic analysis.

Plant species	Accession ID	Source
Anethum foeniculum	GCA_003724115.2	NCBI
Apium graveolens	GCA_009905375.1	NCBI
Heracleum sosnowskyi	GCA_030848705.1	NCBI
Saposhnikovia divaricata	GCA_036435485.1	NCBI
Angelica sinensis	CNP0003198	China National GeneBank DataBase (CNGBdb)
Peucedanum praeruptorum	PRJNA910498	https://figshare.com/articles/dataset/Peucedanum_praeruptorum_genome_assembly_and_gene_annotations/21743984/1

Table S10. Reported UbiA PTs used in phylogenetic analysis.

UbiA PT in primary metabolism	Plant species	Accession No.
**ABC4s in phylloquinone biosynthesis**		
AtABC4	*Arabidopsis thaliana*	NP_001117518.1
ZmABC4	*Zea mays*	NP_001152170.1
**ATGs in chrolophyll biosynthesis**		
AtATG4	*Arabidopsis thaliana*	NP_190750.1
ZmATG4	*Zea mays*	NP_001142204.1
**COX10s in heam *a* biosynthesis**		
AtCOX10	*Arabidopsis thaliana*	NP_566019.1
ZmCOX10	*Zea mays*	AFW89544.1
**PPTs in ubiquinone biosynthesis**		
AtPPT1	*Arabidopsis thaliana*	NP_567688
ZmPPT	*Zea mays*	AQL04517.1
**VTE2-1s in tocopherol biosynthesis**		
AtVTE2-1	*Arabidopsis thaliana*	NP_849984.1
ZmVTE2-1	*Zea mays*	ACG45339.1
**VTE2-2s in plastoquinone biosynthesis**		
AtVTE2-2	*Arabidopsis thaliana*	NP_001154609.1
ZmVTE2-2	*Zea mays*	NP_001146703.1
**Apiaceae-specific PTs**		
AkPT1	*Angelica keiskei*	BCH36133.1
PcPT	*Petroselinum crispum*	BAO31627.1
PsPT1	*Pastinaca sativa*	AJW31563.1

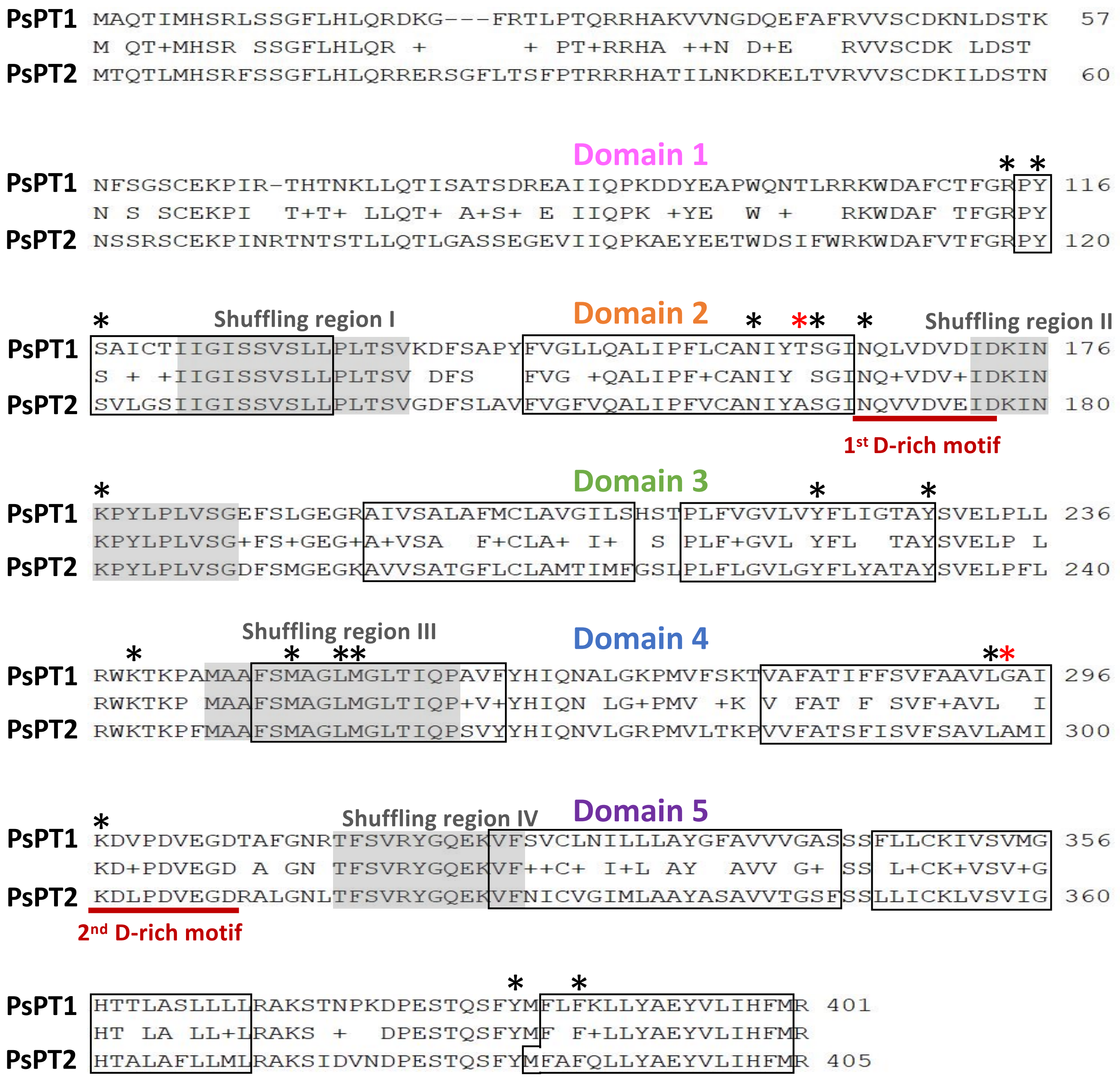

Fig. S1. Polypeptide sequence alignment of PsPT1 and PsPT2.
Gray shadows, shuffling regions; red underlines, 1st and 2nd D-rich motifs; boxes, transmembrane regions predicted by TMHMM 2.0. Amino acids close to substrates in 3D models of PsPT1 and PsPT2 (Supplementary Fig. S9) are marked with asterisks with red asterisks representing mismatches between PsPT1 and PsPT2.

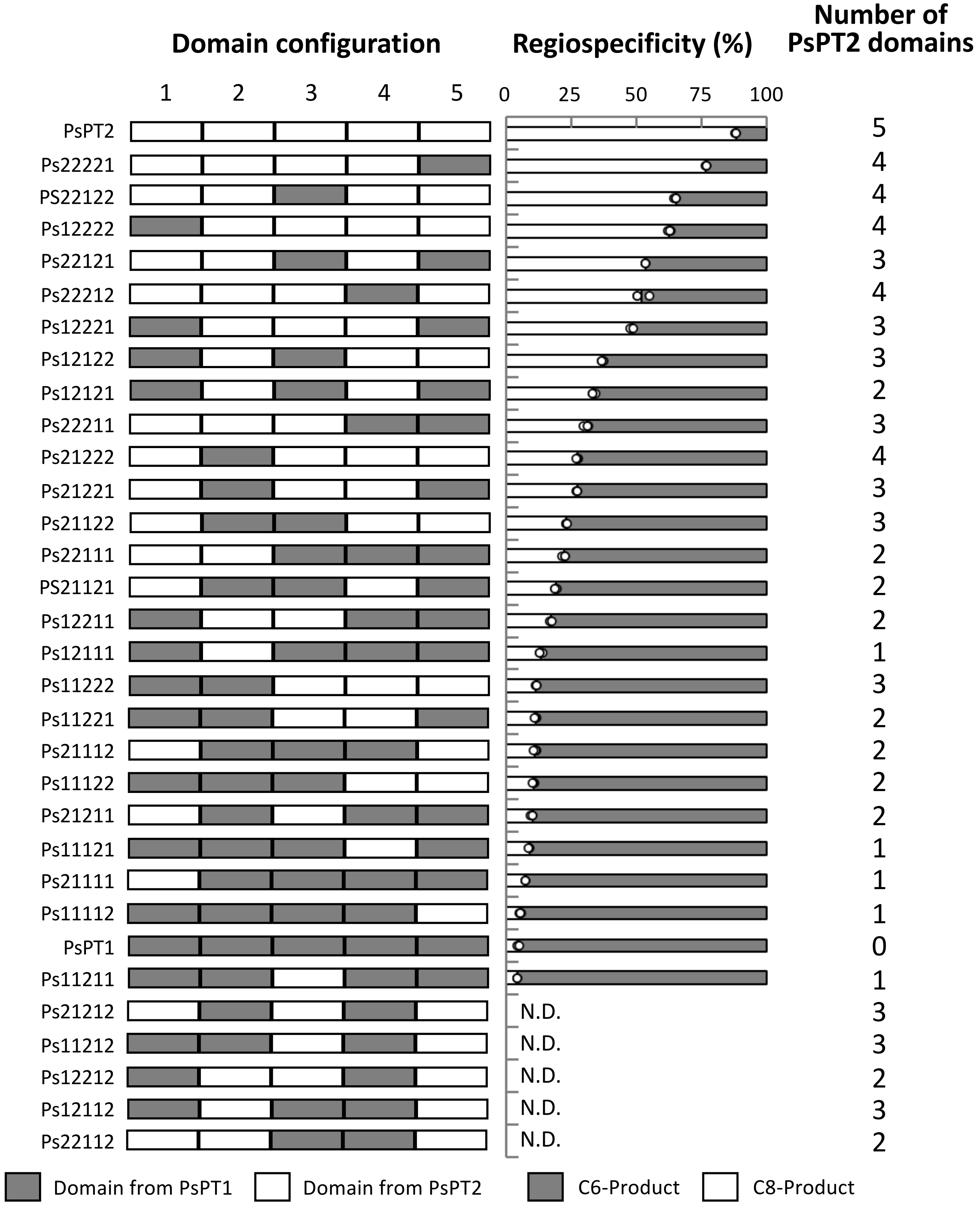

Fig. S2. The regiospecificity of 30 domain-swapped chimeric enzymes of PsPT1 and PsPT2.

The regiospecificity of each enzyme is determined by triplicate independent reactions (n = 3). N.D., not detected. Domain configuration: gray bars, domains from PsPT1; white bars, domains from PsPT2. Regiospecificity: gray bars, prenylation product at the C6-position; white bar, prenylation product at the C8-position.

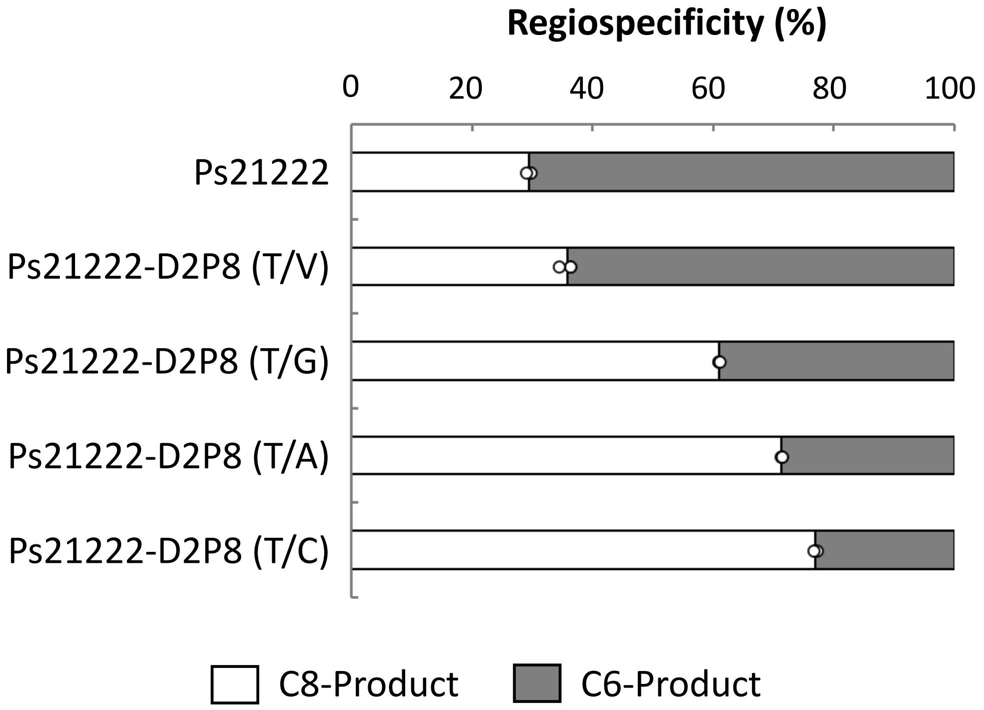

Fig. S3. Introduction of point mutations into the D2P8 position of Ps21222.

The regiospecificity was determined by triplicate independent reactions. The UDT activity was not detected for Ps21222-D2P8 mutants with F, Y, P, K, S, R, N, L, Q, W, H, M, D, E, or I.

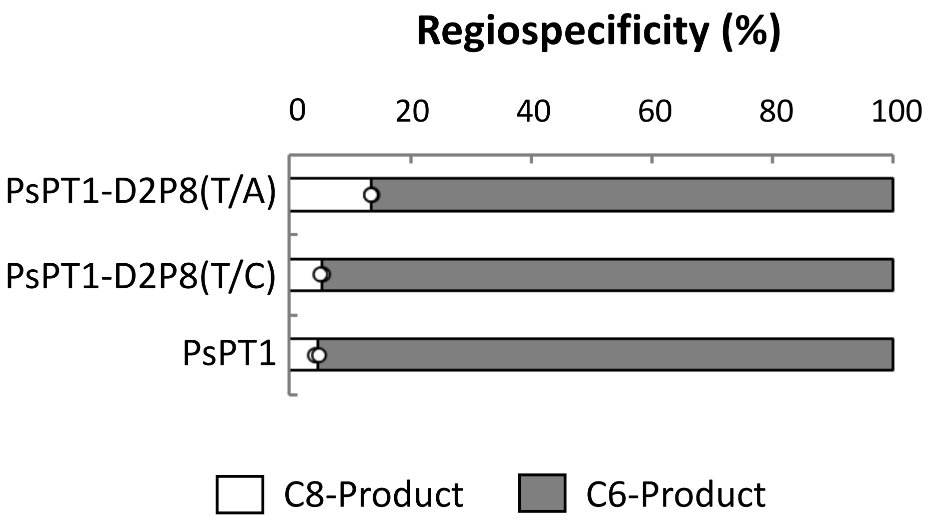

Fig. S4. Introduction of point mutations of amino acids at position D2P8 of PsPT1.

The regiospecificity was determined by three independent reactions.

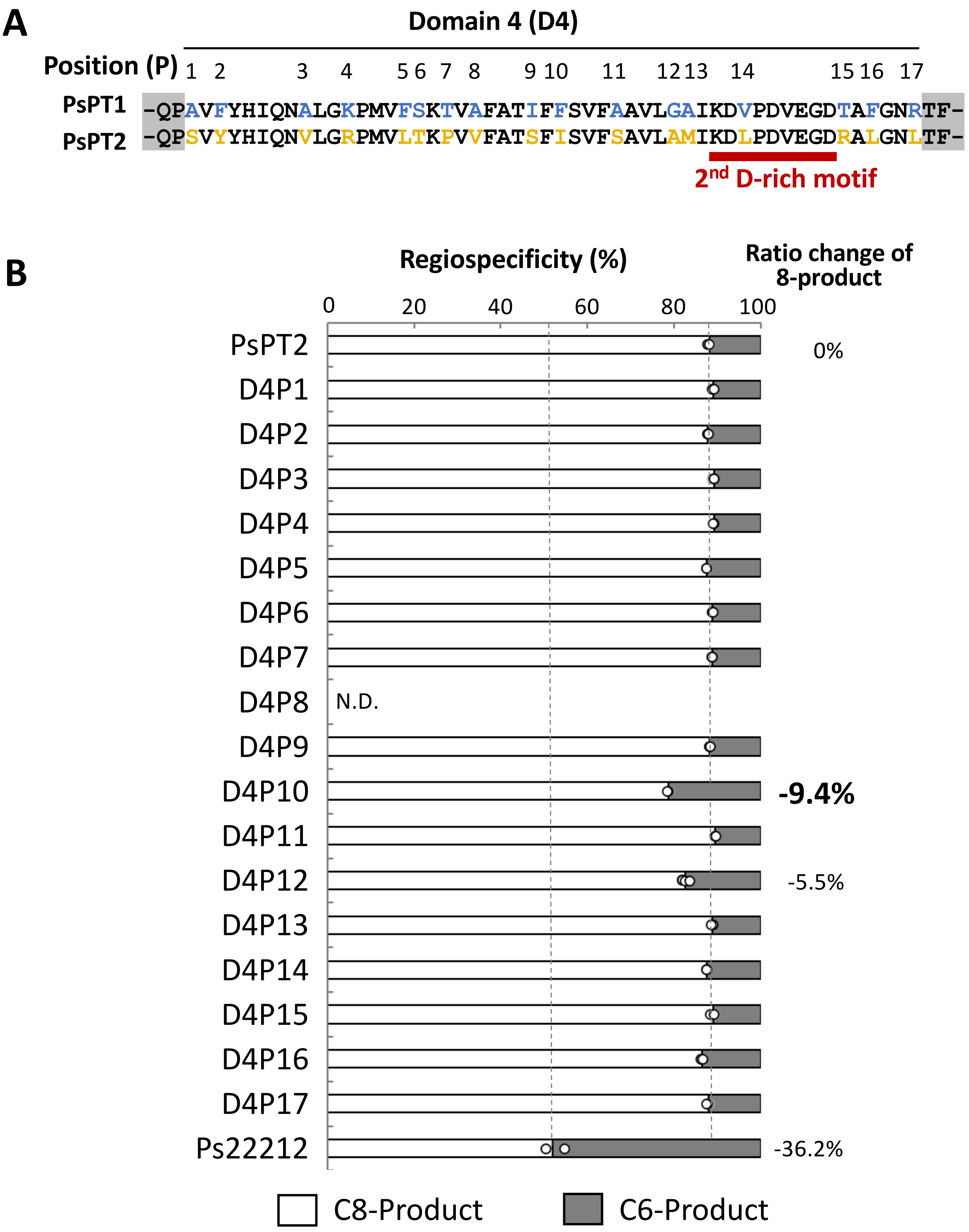

Fig. S5. Introduction of point mutations of amino acids from PsPT1 into the sequence of domain 4 of PsPT2.

A: There are 17 mismatch sites in domain 4 between PsPT1 and PsPT2.

B: Point mutations were introduced individually into PsPT2 at 17 mismatch sites using the amino acids of PsPT1 to generate 17 point mutants and their regiospecificities were confirmed by independent triplicate experiments. N.D., not detected. White, prenylated product at the C8-position. Gray, prenylated product at the C6-position. Data from PsPT2 and Ps22212 in Fig. S2 are shown as controls.

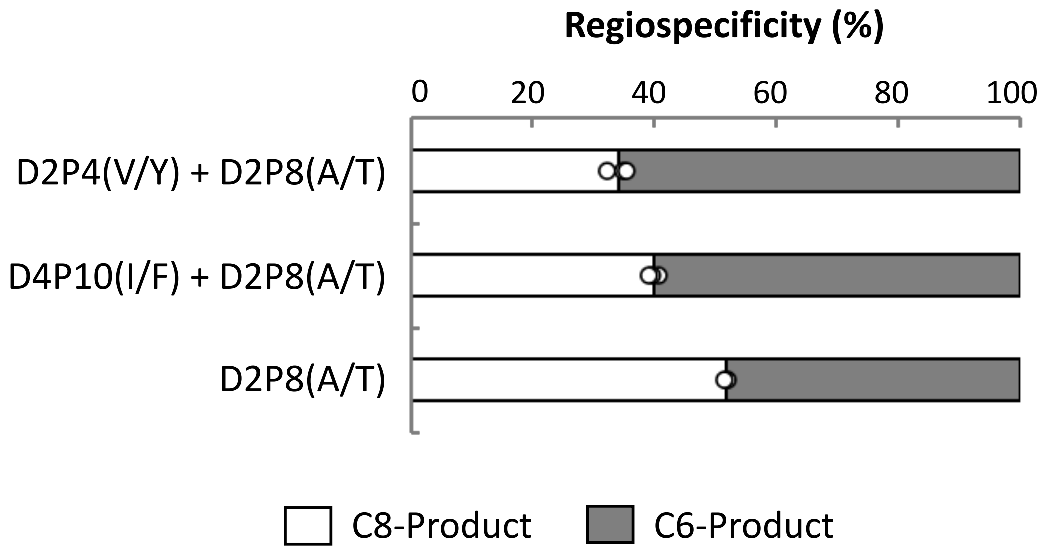

Fig. S6. Introduction of point mutations of amino acids from PsPT1 into the sequence of PsPT2-D2P8(A/T).

The regiospecificity was determined by three independent reactions.

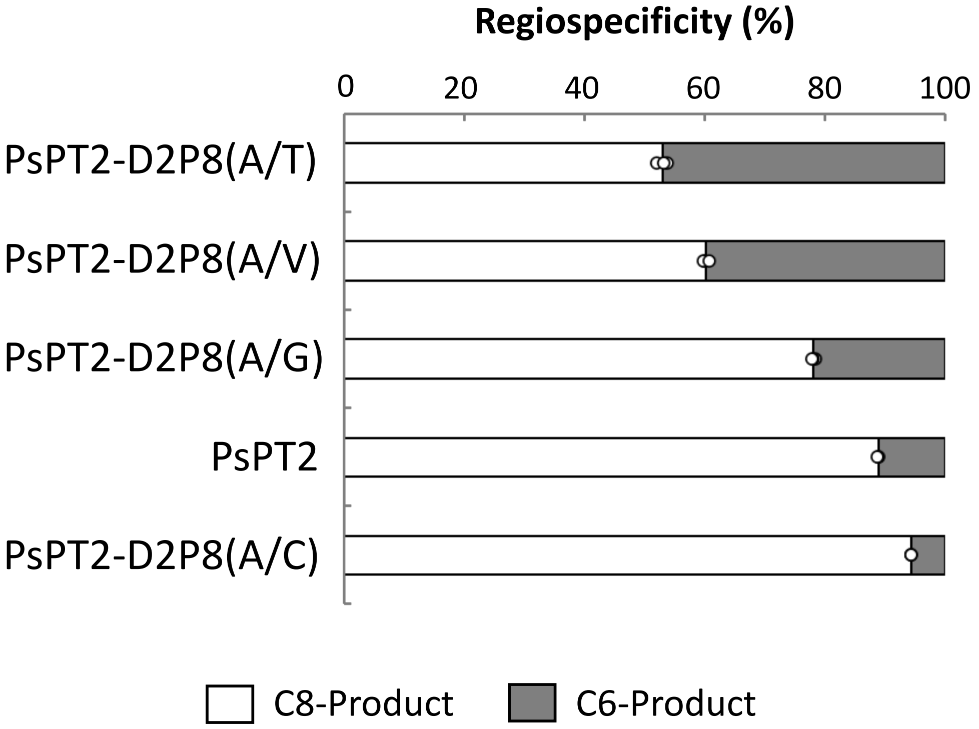

Fig. S7. Introduction of point mutations of amino acids at position D2P8 of PsPT2.
The regiospecificity was determined by three independent reactions.

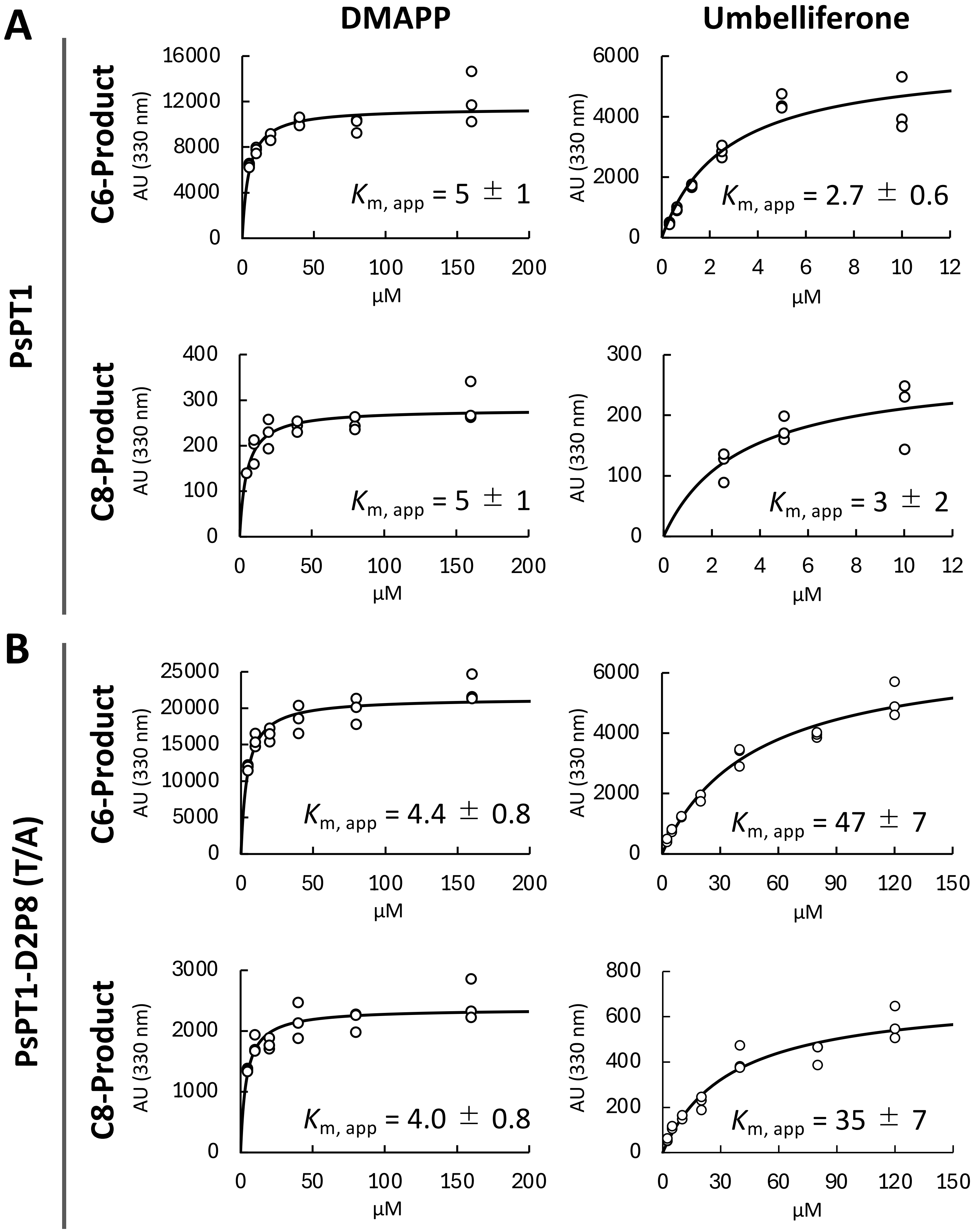

Fig. S8. Kinetic analysis of PsPTs and their mutants.

C

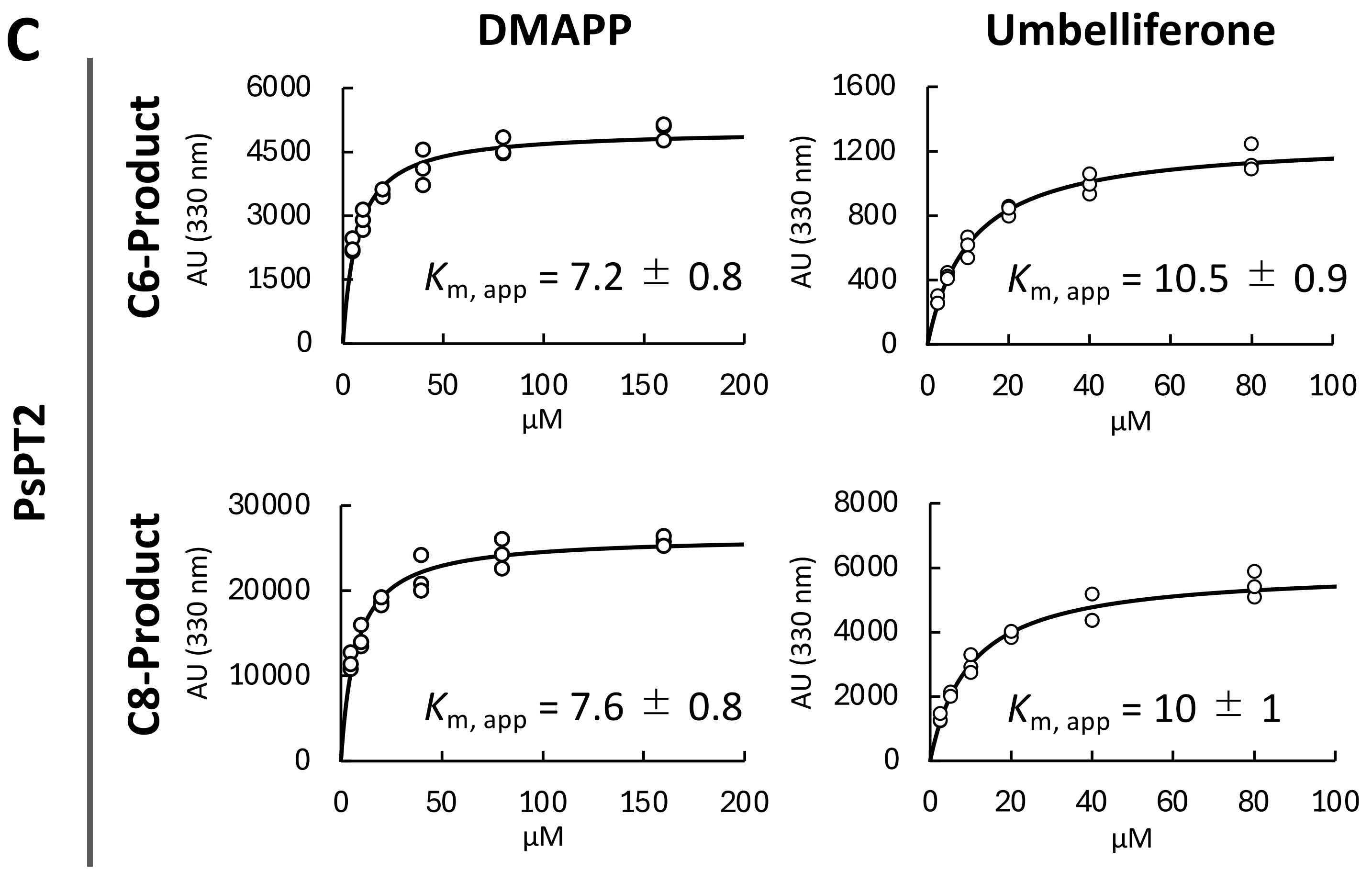

D

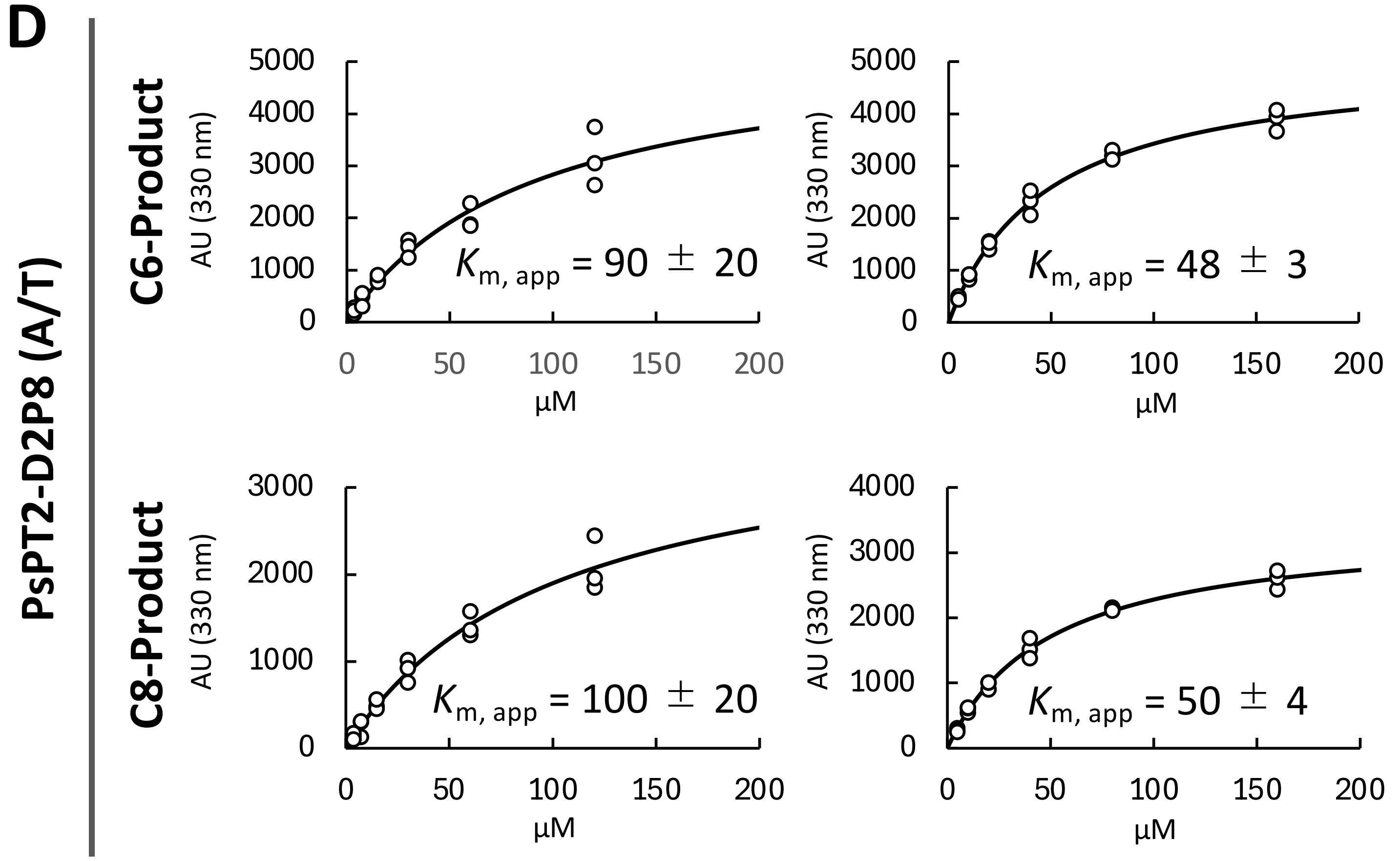

Fig. S8. Kinetic analysis of PsPTs and their mutants. -*continued*

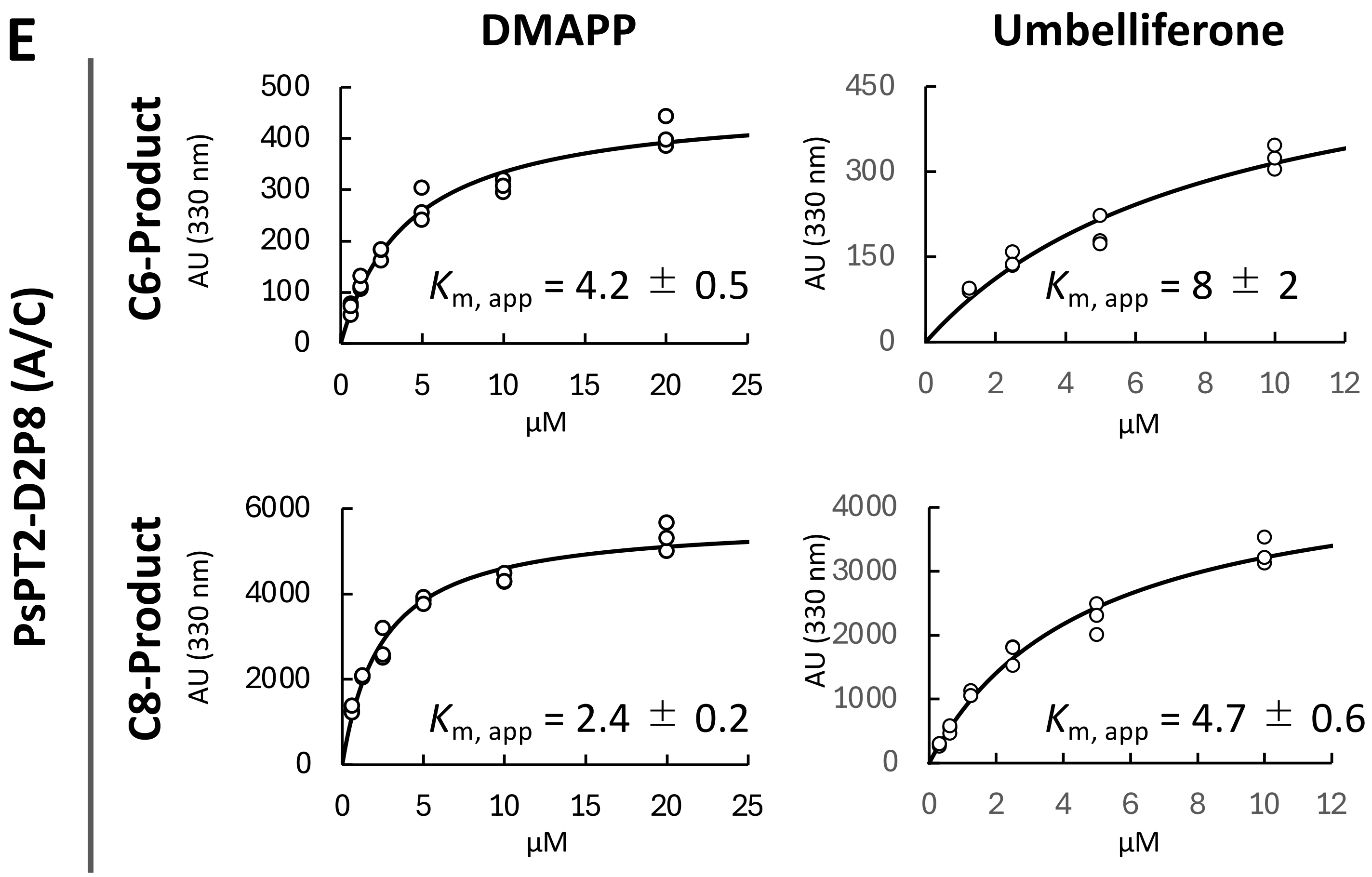

Fig. S8. Kinetic analysis of PsPTs and their mutants. -*continued*
Enzymatic activities of PsPT1 (**A**), PsPT1-D2P8 (T/A) (**B**), PsPT2 (**C**), PsPT2-D2P8 (A/T) (**D**), and PsPT2-D2P8 (A/C) (**E**) in reactions with different substrate concentrations (n = 3). Fitting curves were plotted using Sigmaplot 14.5. Apparent *K*m values are expressed as mean ± standard error.

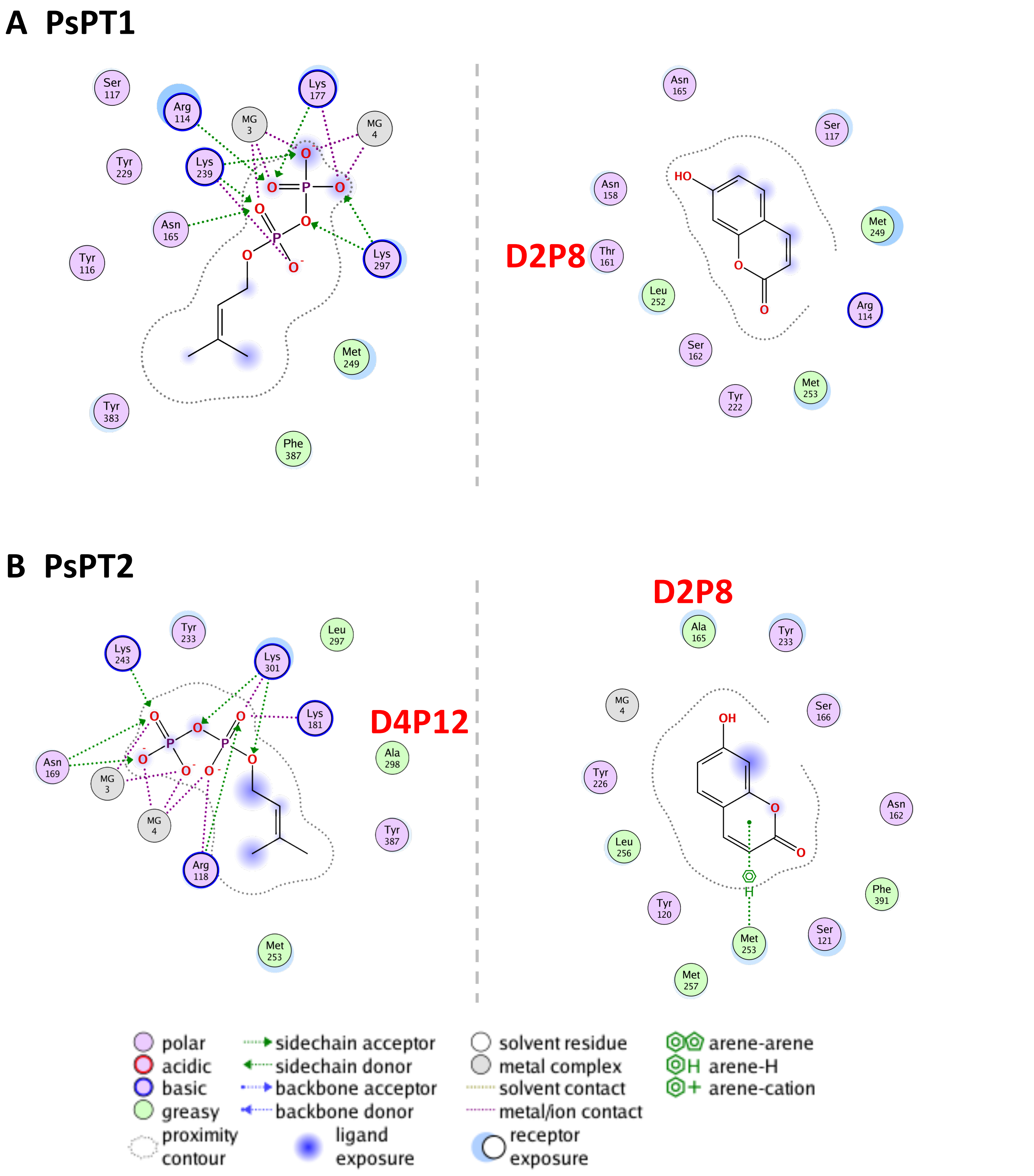

Fig. S9. Amino acid residues near to substrates in 3D models of PsPT1 and PsPT2.

Han et al.

C

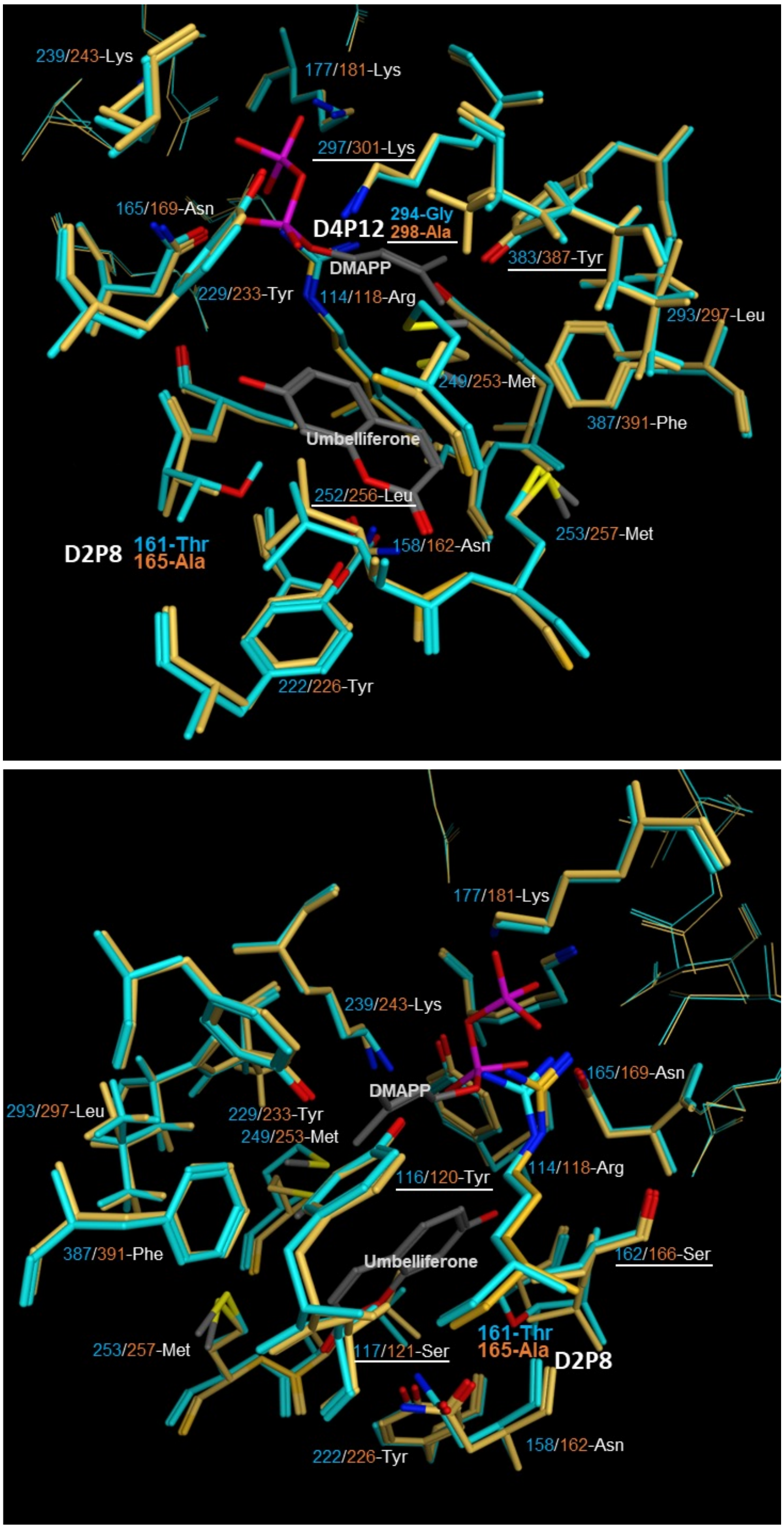

Fig. S9. Amino acid residues near to substrates in 3D models of PsPT1 and PsPT2. *-continued*

Amino acid residues close to DMAPP and umbelliferone are for PsPT1 (A) and PsPT2 (B). These residues are also highlighted in Fig S2. Among these amino acids, two mismatch positions between PsPT1 and PsPT2 correspond to D2P8 and D4P12. (C) Superimposed images of 3D models of PsPT1 (light blue) and PsPT2 (yellow). Images are taken from two angles to capture the orientations of all residues shown in (A) and (B). Underlined residues are found in only one of the images.

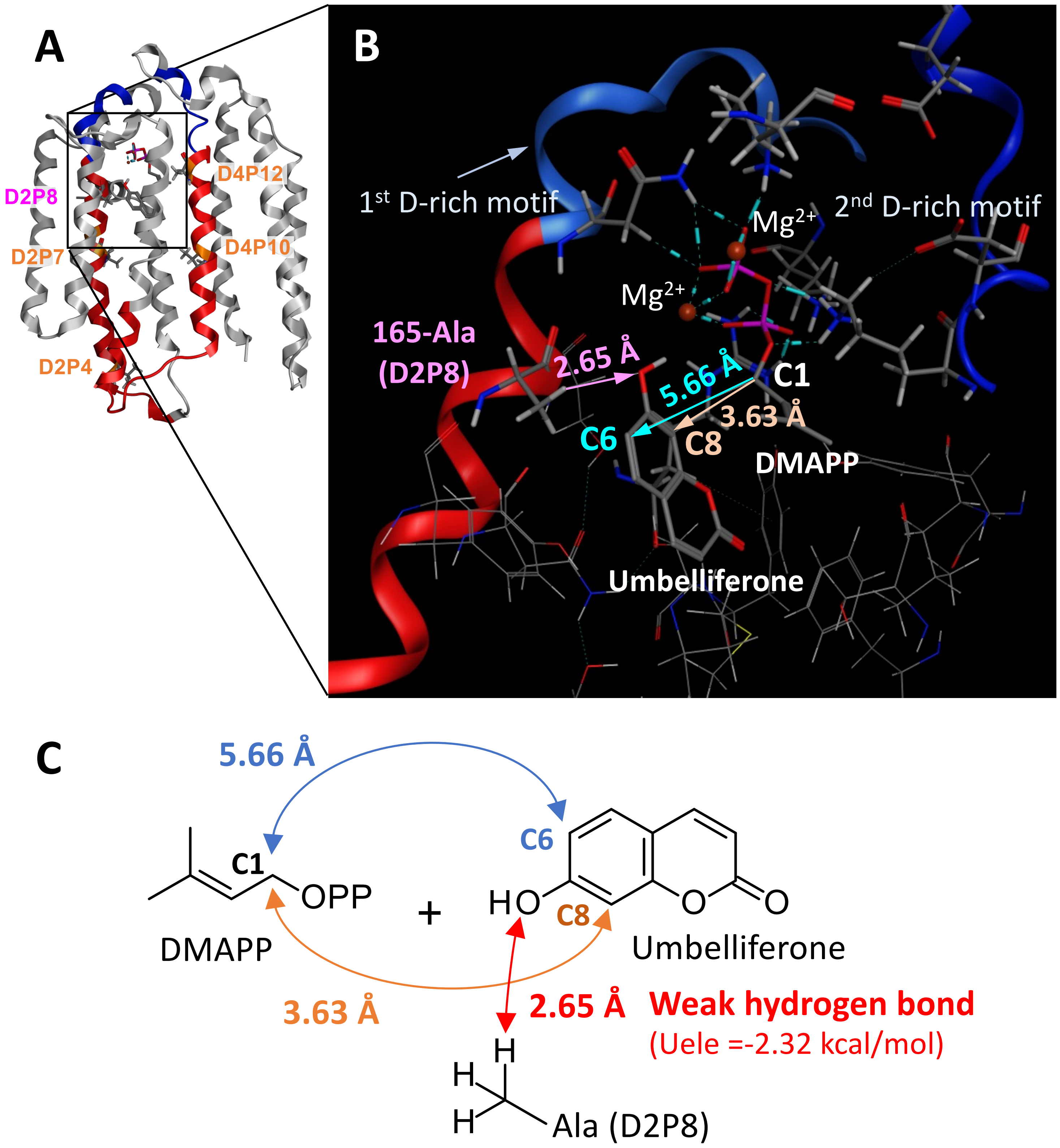

Fig. S10. A 3D model of substrate-bound PsPT2.

The overall structure (**A**) and the catalytic pocket (**B**) of the most appropriate ligand configuration obtained by simulation analysis (Table S2). The distance between C1 of DMAPP and C6 of umbelliferone is related to the U6DT activity, and the distance between C1 of DMAPP and C8 of umbelliferone is related to the U8DT activity. **C:** Model diagram of the interaction between the D2P8 position (165-Ala) of PsPT2 and umbelliferone. A weak hydrogen bond (Uele = -2.32 kcal/mol) between umbelliferone and 165-Ala is proposed.

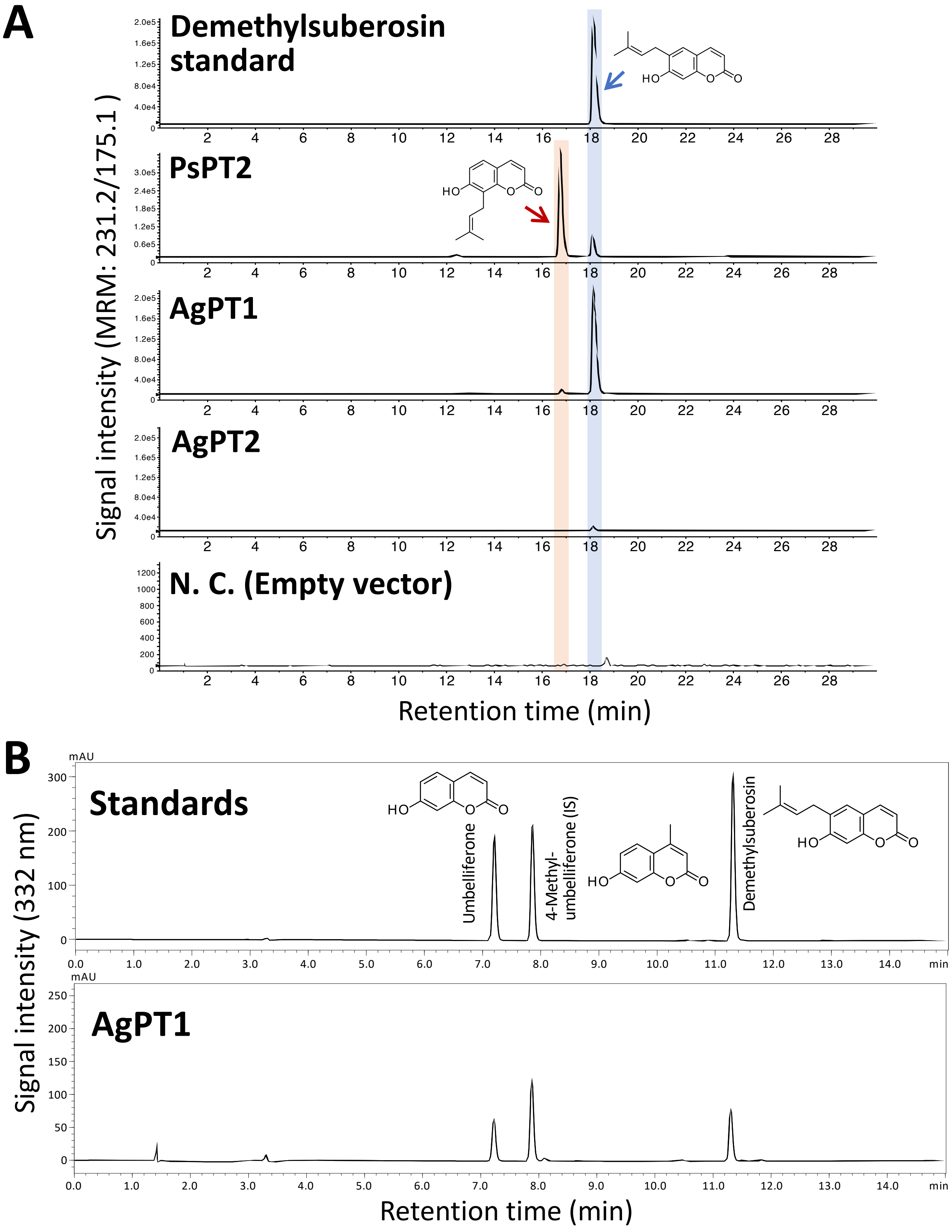

Fig. S11. Enzyme activities of AgPT1 and AgPT2 from *Anethum graveolens*.
A: MS chromatograms of UDT reaction mixtures. Multiple Reaction Monitoring (MRM) (parent ion, m/z = 231.2; fragment ion, m/z=175.1) was performed in the positive ion mode. Retention times of demethylsuberosin (C6-product) and osthenol (C8-product) are shown in PsPT2 and demethylsuberosin standard. N. C., negative control.
B: UV chromatograms of UDT reaction mixtures at 332 nm.

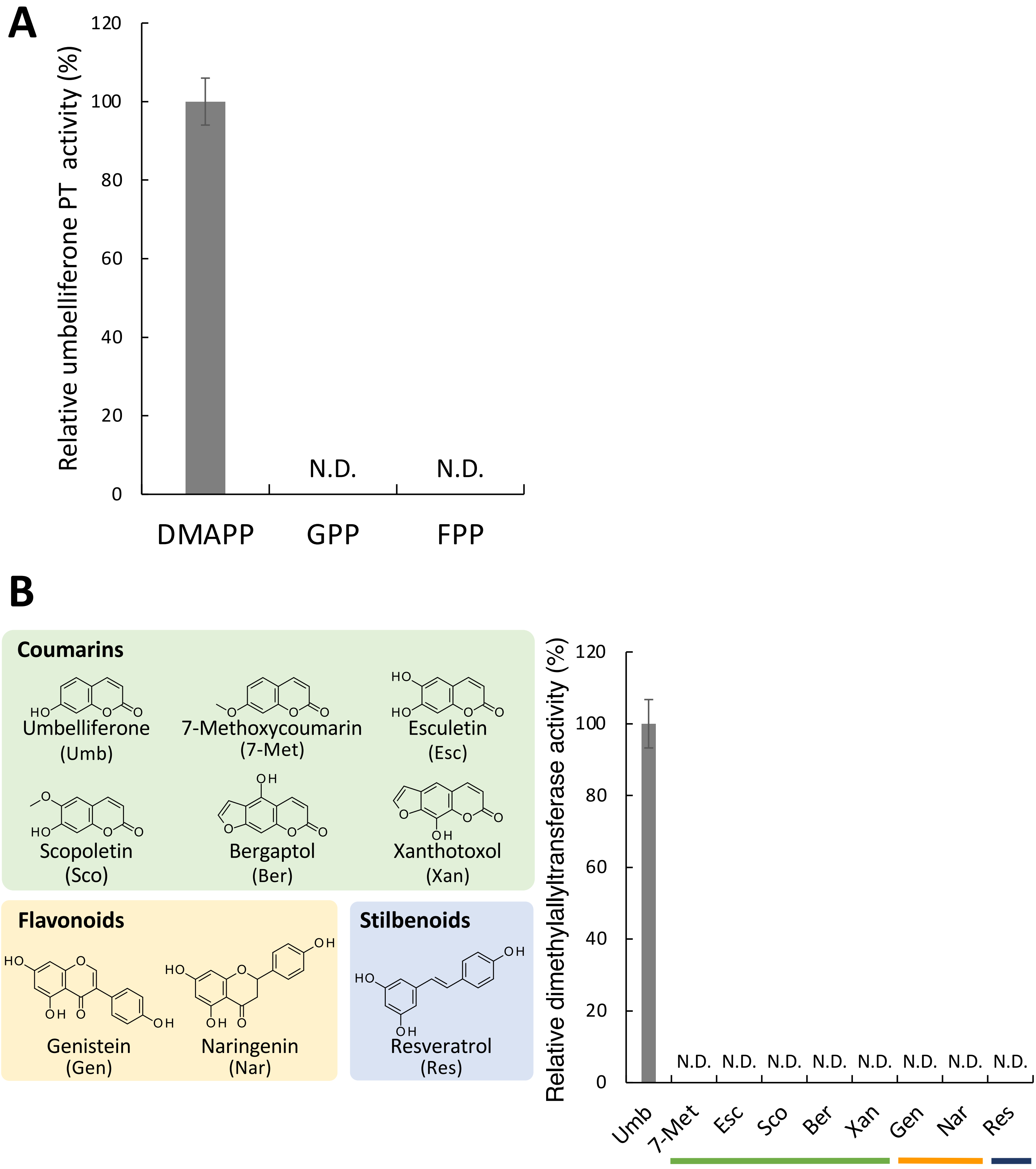

Fig. S12. Substrate specificity of AgPT1.

A: Prenyl donor specificity using umbelliferone as a prenyl acceptor.

B: Prenyl acceptor specificity using DMAPP as prenyl donor. Data are expressed as mean ± standard error (n = 3).

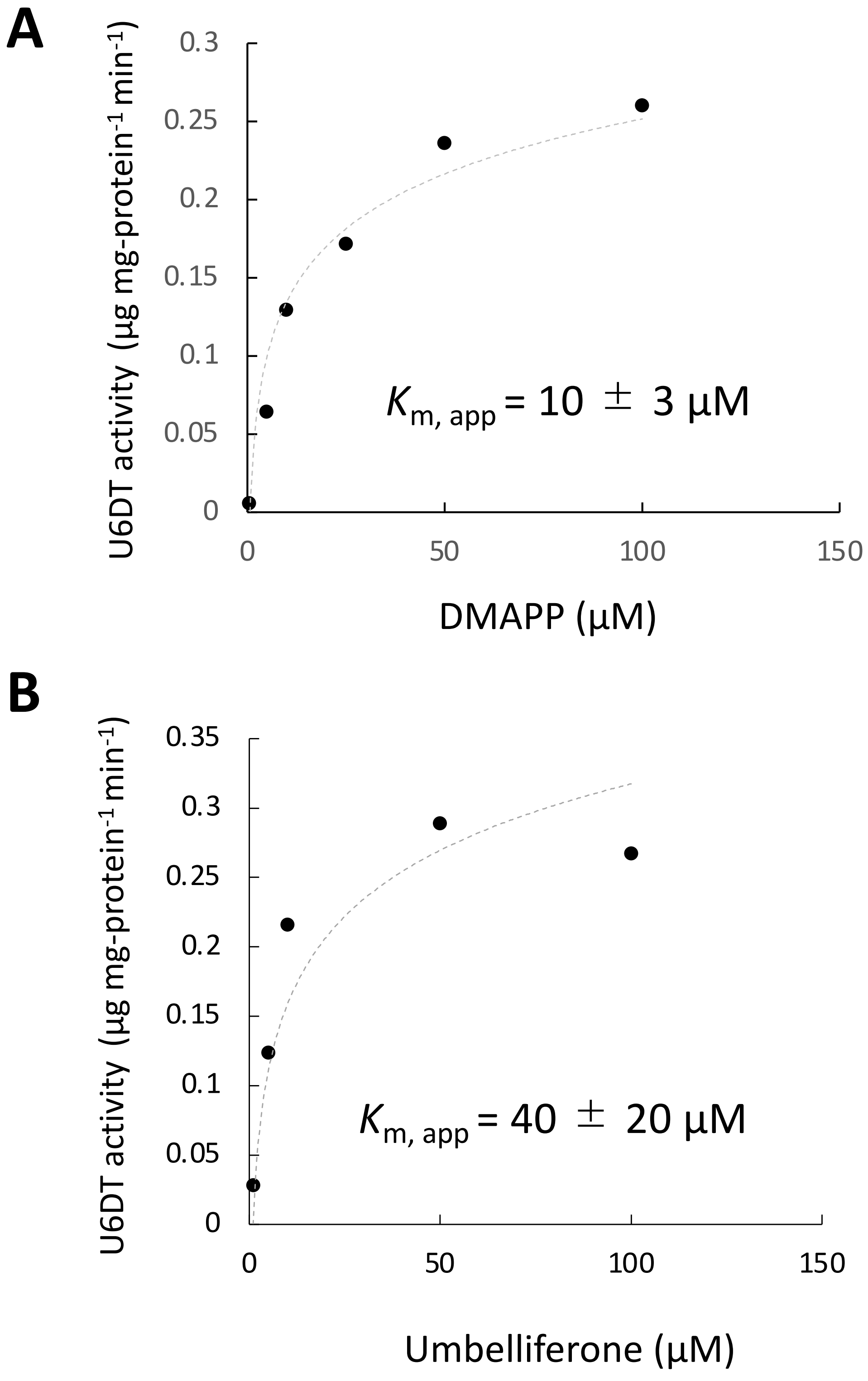

Fig. S13. Apparent K_m values of AgPT1.

Apparent K_m values for DMAPP (**A**) and umbelliferone (**B**) of AgPT1. Data are presented as mean ± standard error (n = 3) with representative fitted curves.

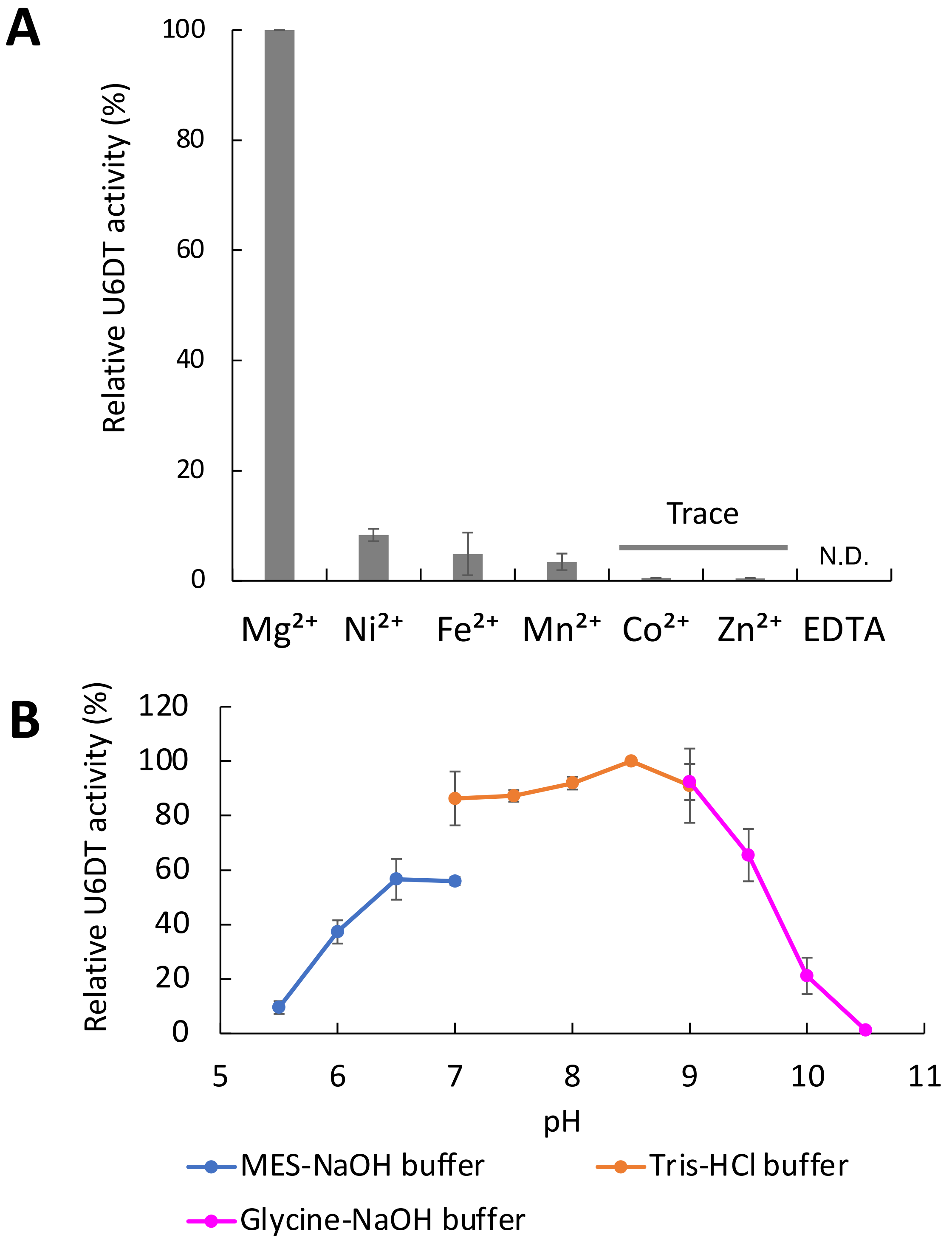

Fig. S14. Divalent cation requirement and pH optimum of AgPT1.
A: Divalent cation requirement of AgPT1. **B:** pH dependence of AgPT1. Data are expressed as mean ± standard error (n = 3).

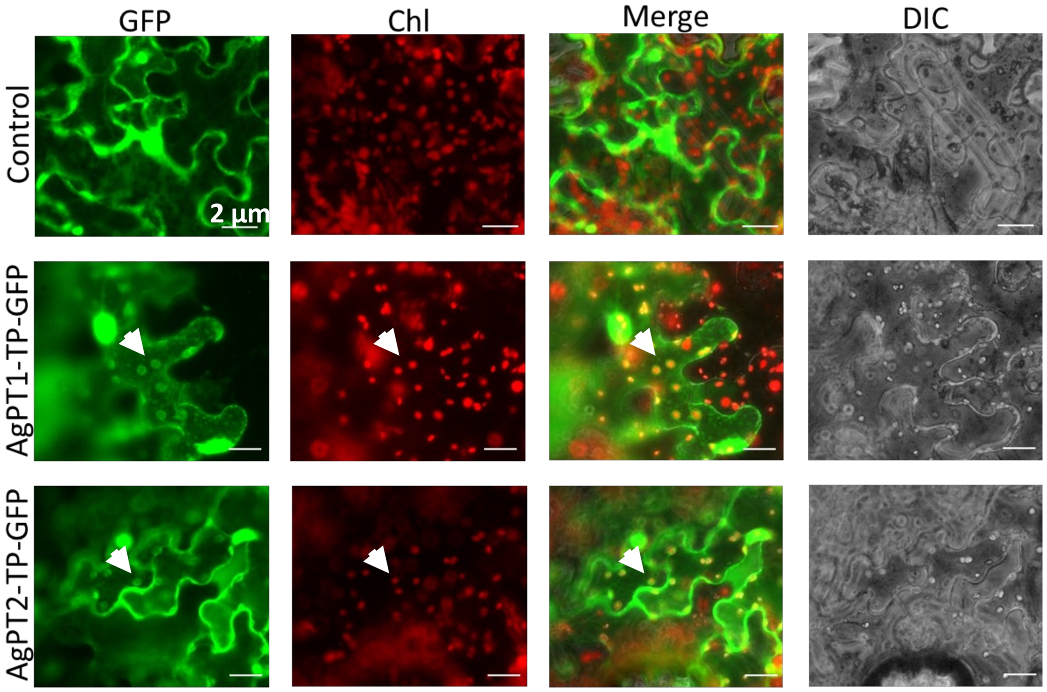

Fig. S15. Subcellular localization of AgPT1 and AgPT2.
AgPT1-TP-GFP and *AgPT2-TP-GFP* were introduced into *N. benthamiana* leaves using the pEAQ-HT-DEST1 vector, which allows high production of recombinant proteins using the Cowpea Mosaic Virus hypertranslational expression system. Images were obtained by fluorescence microscopy. Experimental details are described in Materials and Methods of Supplementary data.

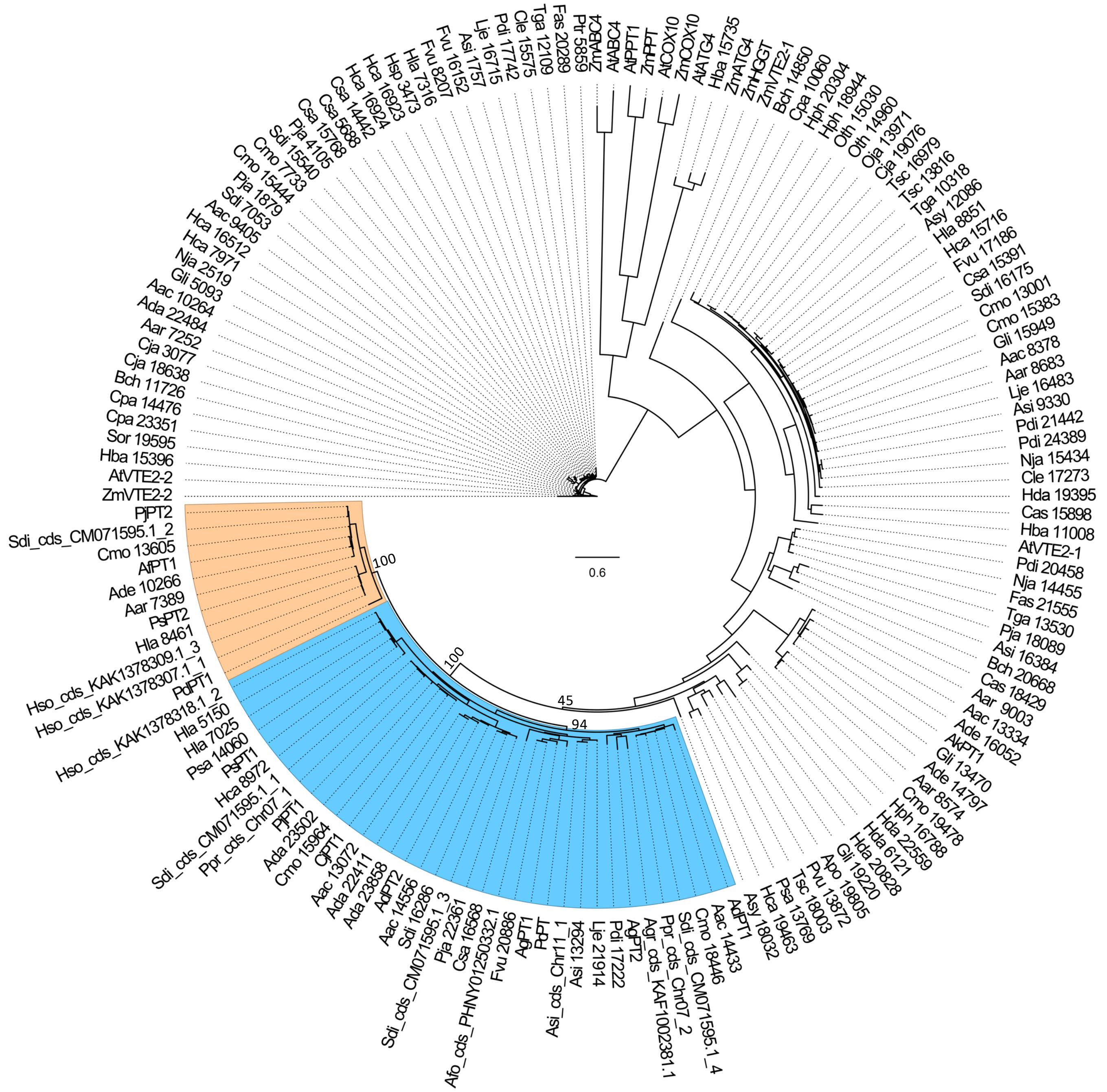

Fig. S16. A phylogenetic tree of apiaceous UbiA PTs.

UbiA PT polypeptide sequences of Apiaceae plants were aligned with primary UbiA PTs from *Arabidopsis thaliana* and *Zea mays* using MAFFT alignment. A phylogenetic tree was generated with RAxML, employing the GAMMA JTT model and 1000 bootstrap replicates. Clades containing U6DT and U6DT-like sequences and U8DT and U8DT-like sequences were highlighted by blue and orange backgrounds, respectively. The scale bar represents an amino acid substitution rate per site of 0.6. Bootstrap values were shown for nodes closed to the U6DT and U8DT clades. Information on UbiA PTs in the tree were shown in Supplementary Fig. S10 and S18. Apiaceous PT sequences collected from transcriptome data and genomic data are described as species plus numbers (e.g., Asi 13294) and species_cds_sequence ID (e.g., Asi_cds_Chr11_1), respectively.

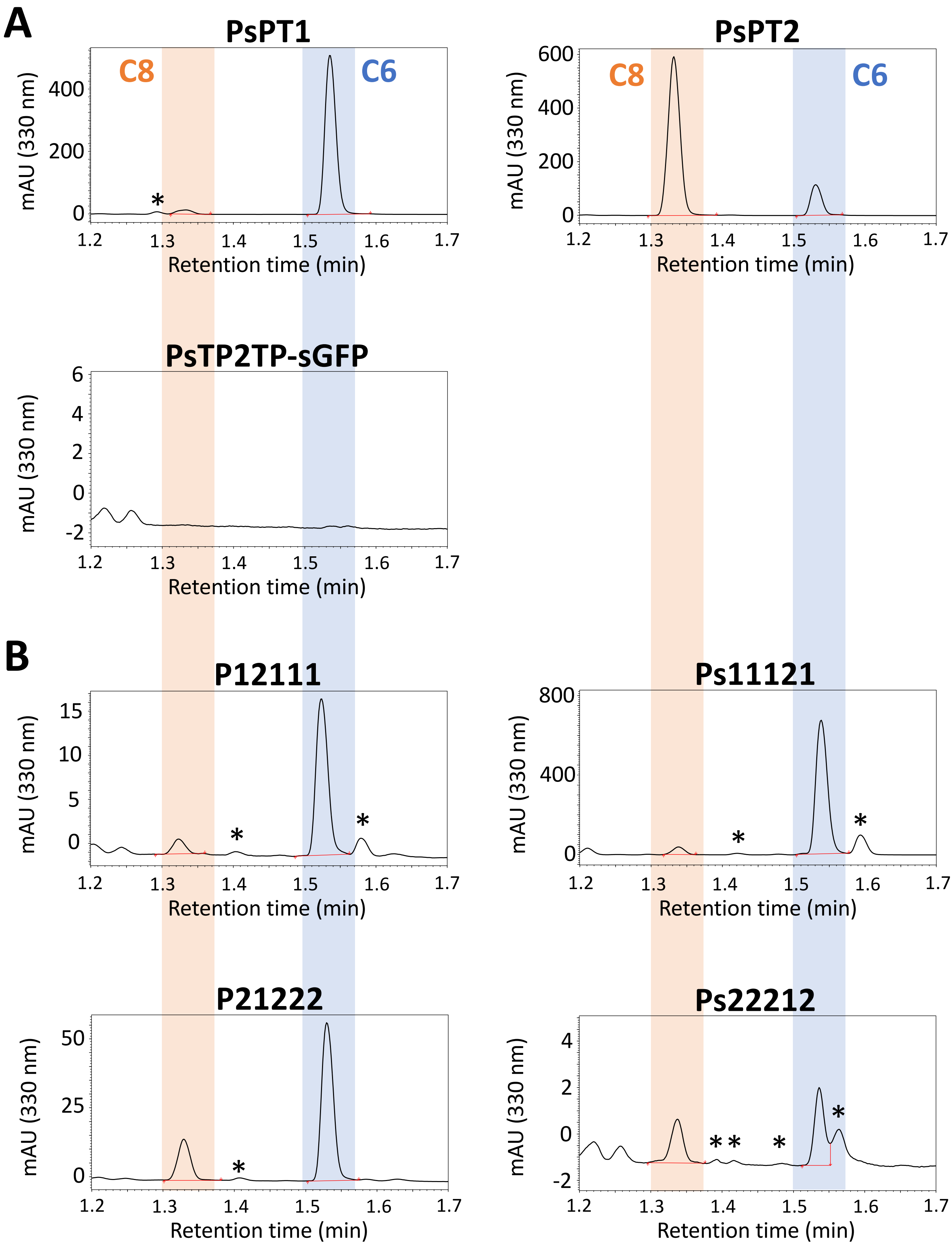

Fig. S17. Quantification of osthenol and demethylsuberosin produced by representative enzymes.

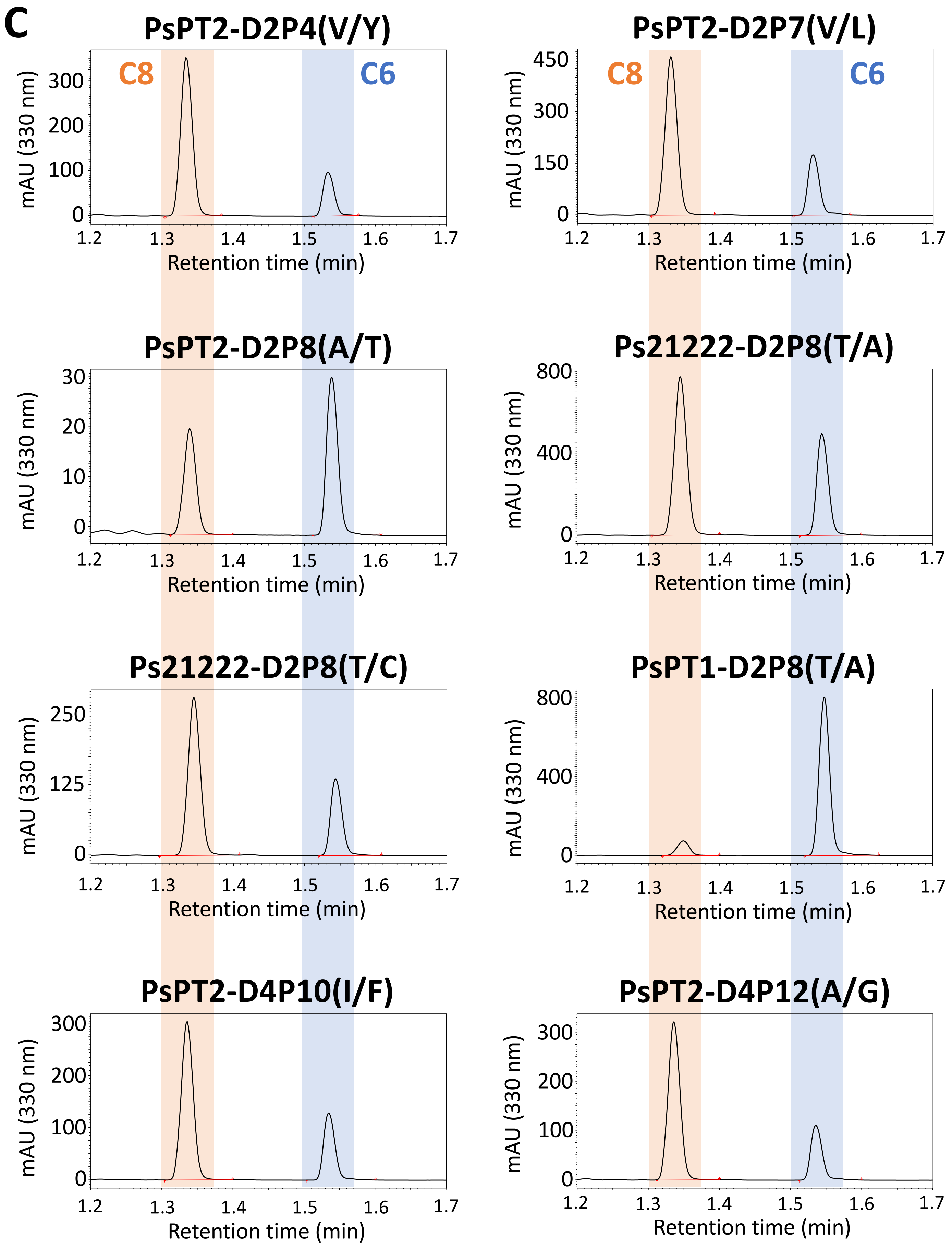

Fig. S17. Quantification of osthenol and demethylsuberosin produced by representative enzymes.-*continued.*

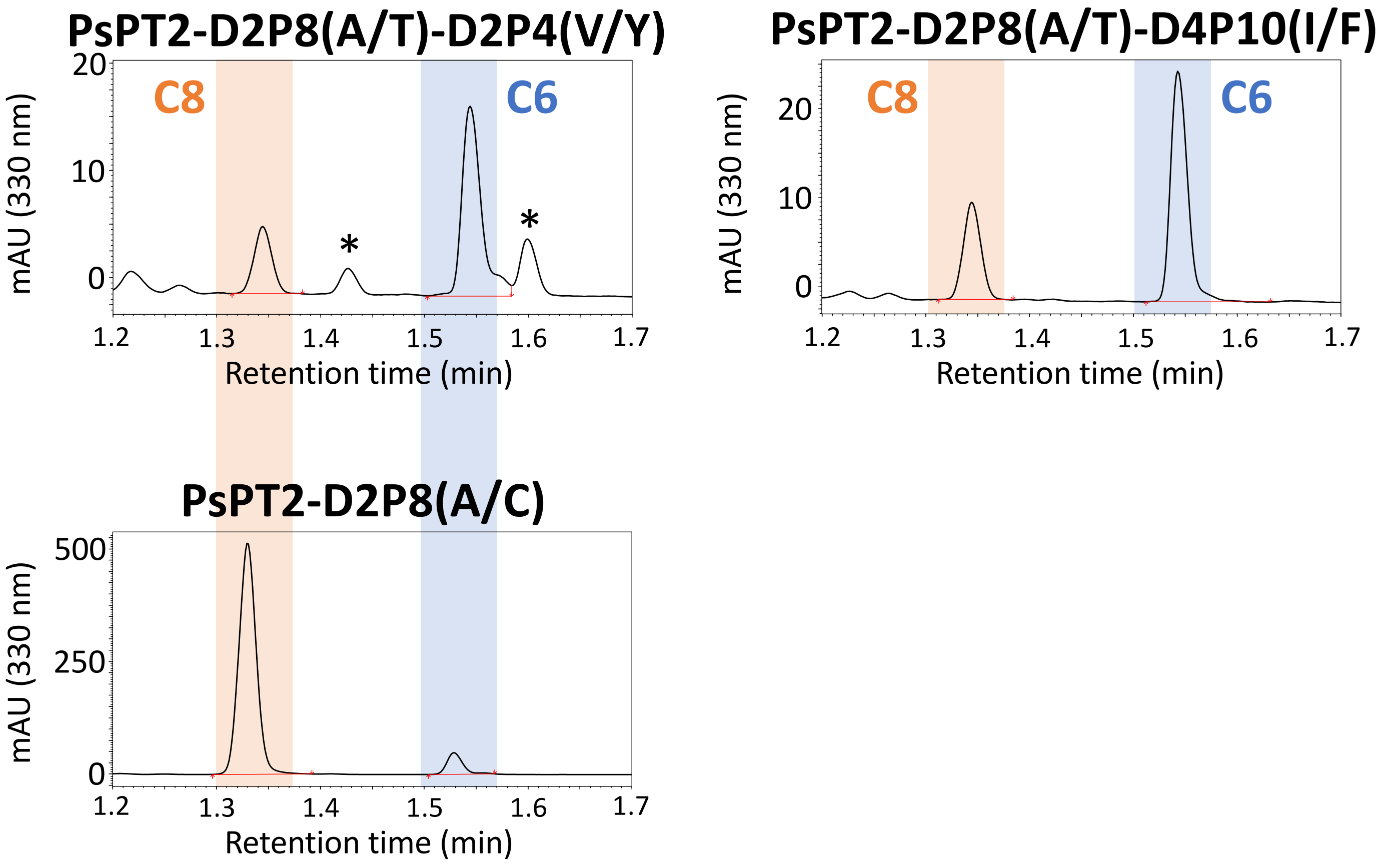

Fig. S17. Chromatograms used for quantification of osthenol and demethylsuberosin produced by representative enzymes.-*continued.*
UV chromatograms at 330 nm of UDT reaction mixtures of PsPT1, PsPT2 and PsPT2TP-sGFP as negative control (A), domain-swapped mutants (B) and point mutants (C) are shown with baselines for quantification of osthenol (C8-product) and demethylsuberosin (C6-product). Peaks from these reaction products are highlighted. Asterisks indicate unidentified peaks. Only for Ps22212 the microsomal fraction was used as the crude enzyme because the UDT activity of Ps22212 was too low when the simplified method was applied to the enzyme assay.
